# Analysis of FOXP3^+^ Regulatory T Cells That Display Apparent Viral Antigen Specificity during Chronic Hepatitis C Virus Infection

**DOI:** 10.1371/journal.ppat.1000707

**Published:** 2009-12-24

**Authors:** Shuo Li, Stefan Floess, Alf Hamann, Silvana Gaudieri, Andrew Lucas, Margaret Hellard, Stuart Roberts, Geza Paukovic, Magdalena Plebanski, Bruce E. Loveland, Campbell Aitken, Simon Barry, Louis Schofield, Eric J. Gowans

**Affiliations:** 1 Macfarlane Burnet Institute for Medical Research and Public Health, Melbourne, Victoria, Australia; 2 Experimental Rheumatology, Charité University Medicine Berlin, Berlin, Germany; 3 Centre for Clinical Immunology and Biomedical Statistics, Perth, Western Australia, Australia; 4 School of Anatomy and Human Biology and Centre for Forensic Science, University of Western Australia, Perth, Western Australia, Australia; 5 Department of Gastroenterology, Alfred Hospital, Melbourne, Victoria, Australia; 6 Department of Immunology, Monash University, Melbourne, Victoria, Australia; 7 The Women's and Children's Health Research Institute, Adelaide, South Australia, Australia; 8 The Walter and Eliza Hall Institute of Medical Research, Parkville, Victoria, Australia; 9 Department of Microbiology, Monash University, Melbourne, Victoria, Australia; The Wistar Institute, United States of America

## Abstract

We reported previously that a proportion of natural CD25^+^ cells isolated from the PBMC of HCV patients can further upregulate CD25 expression in response to HCV peptide stimulation *in vitro*, and proposed that virus-specific regulatory T cells (Treg) were primed and expanded during the disease. Here we describe epigenetic analysis of the FOXP3 locus in HCV-responsive natural CD25^+^ cells and show that these cells are not activated conventional T cells expressing FOXP3, but hard-wired Treg with a stable FOXP3 phenotype and function. Of ∼46,000 genes analyzed in genome wide transcription profiling, about 1% were differentially expressed between HCV-responsive Treg, HCV-non-responsive natural CD25^+^ cells and conventional T cells. Expression profiles, including cell death, activation, proliferation and transcriptional regulation, suggest a survival advantage of HCV-responsive Treg over the other cell populations. Since no Treg-specific activation marker is known, we tested 97 NS3-derived peptides for their ability to elicit CD25 response (assuming it is a surrogate marker), accompanied by high resolution HLA typing of the patients. Some reactive peptides overlapped with previously described effector T cell epitopes. Our data offers new insights into HCV immune evasion and tolerance, and highlights the non-self specific nature of Treg during infection.

## Author Summary

Hepatitis C virus persistently infects ∼3% of the world population, leading to life threatening liver diseases and liver failure. It is not well understood why the human immune system often fails to clear the virus, although it is likely multi-factorial. It is accepted that effector T cells are critical for clearing infections, but their function can be suppressed by the somewhat elusive regulatory T cells. Our hypothesis, supported by new data, is that a proportion of the regulatory T cells are specifically stimulated by the virus and that these cells are a stable cell population. We find evidence that these suppressive cells have a distinct set of genes activated and importantly might have a survival advantage over effector T cells, which helps to explain why natural regulatory T cells may influence the outcome of HCV infection. We propose that the new information provides a better explanation of chronic HCV infection and will let us focus on the key experiments to test the hypothesis and to design better treatments.

## Introduction

Hepatitis C virus is a small positive sense single stranded RNA virus, which causes persistent infection that leads to cirrhosis, cancer and liver failure. In the acute phase of the infection, the host usually mounts strong CD4^+^ and CD8^+^ T cell responses, but this wanes in the next few months during the transition to persistence (reviewed in reference [1]). Typically, in persistently-infected patients, the frequency of HCV-specific IFNγ-producing effector T cells is low (usually <0.3% of PBMC by ELISPOT) and that of IL2-producing cells is even lower [2]. T cells, particularly CD4^+^ T cells, proliferate poorly in response to HCV antigens [3], although CD8^+^ T cells proliferate slightly better (Li and Gowans, unpublished data). The reason behind the lack of adequate immunity to HCV in human is not well understood, although it is likely to be multi-factorial [1],[4].

IL-10 producing type 1 regulatory T cells (Tr1) may play a role in HCV persistence [5],[6], and more recently, several groups suggested that natural regulatory T cell (Treg, a different type of suppressor cell to Tr1) may be also important [7],[8],[9],[10]. The frequency of circulating CD4^+^CD25^+^ cells (the cell population in which Treg are predominantly contained [11]) in the blood of HCV carriers was higher than in healthy donors and individuals who had resolved the infection [7]. In addition, the percentage of CD4^+^CD25^+^ cells within the infected liver was much higher than in the peripheral blood [8]. (A review of this topic was published recently [12]). One basic property of Treg is that, once activated via the T cell receptor (TCR), they suppress a wide range of immune responses *in vitro* and *in vivo* in a contact-dependent manner [11]. Sugimoto et al. [13] initially showed that depletion of CD25^+^ cells enhanced the proliferation of the remaining PBMC, while Cabrera et al. [7] and several other groups [8]–[10] further showed that CD4^+^CD25^+^ T cells isolated from patients' PBMC could suppress the virus-specific CD8^+^ T-cell response, suggesting that this population contains HCV - specific Treg. The suppressor function of CD4^+^CD25^+^ T cells in response to polyclonal stimuli was further analysed recently in a longitudinal acute phase HCV cohort [10], and it was found that Treg from patients who progressed to persistence were more suppressive than either those from patients who resolved the infection spontaneously or from uninfected healthy donors. In summary, these studies supported the concept that progression from acute to persistent infection is associated with functional changes in the Treg compartment. It is currently unknown, however, to what extend the total Treg pool in HCV-infected individuals is HCV-specific or how Treg react to viral infection as part of the adaptive immune response.

Our group has previously reported [14] that a proportion of natural CD25^+^ cells isolated from the PBMC of HCV patients substantially upregulated CD25 expression in response to HCV peptide stimulation *in vitro*, and we proposed that virus-specific Treg were primed and expanded during the disease. Somewhat disturbingly, the frequency of the hypothetical HCV-specific Tregs far exceeded the well-documented low frequency of IFNγ producing anti-viral effector T cells in chronic infection [1], prompting us to seek more insight to these cells in this study.

## Results

### CD25 expression levels become less homogenous among natural CD25^+^ cells during culture

**Figure 1:**
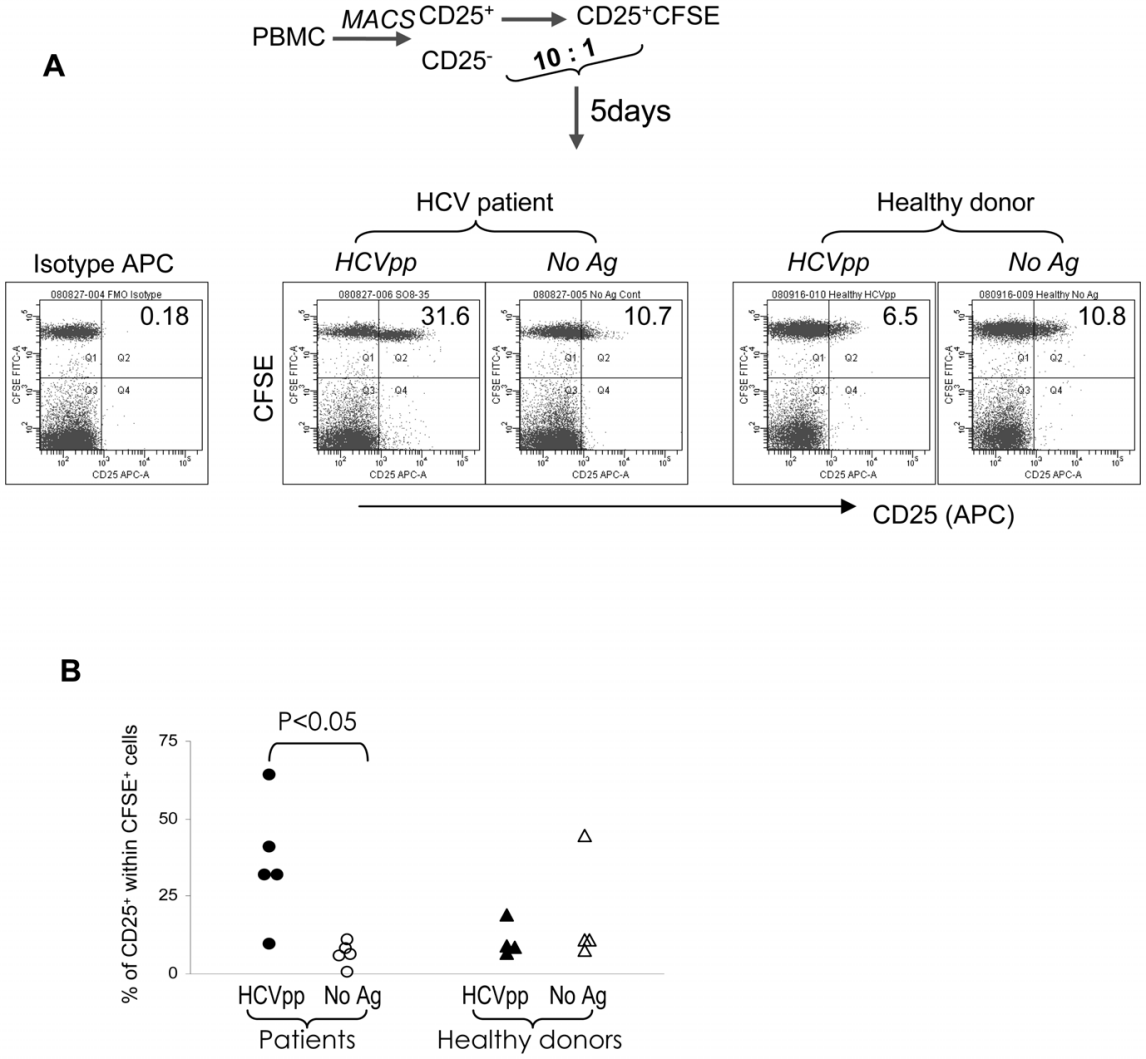
A proportion of natural CD25^+^ from patients, but not healthy donors, can sustain or up-regulate CD25 expression following *in vitro* HCV peptide stimulation. (A) The CFSE-CD25^+^/CD25^−^ co-cultures were stimulated with the HCV peptide pool and analysed on day 5, gating on lymphocytes. The number in the upper right corner indicates the percentage of CD25^+^ cells within the total CFSE^+^ population. (Representative data of N = 5 patients and N = 4 healthy donors). (B) Summary of data from all donors. P value was calculated using student T test.

When the CFSE-CD25^+^/CD25^−^ co-culture from patients was stimulated for 5 days with the HCV peptide pool (pp), CD25 expression on the CFSE^+^ fraction was sustained or up-regulated compared to the non-antigen stimulated control (Figure 1A). This observation is reproducible and statistically significant (p<0.05) (Figure 1B). When healthy donor cells were cultured under the same conditions, the CD25 expression profile in the HCVpp culture was similar to that of the non-antigen control (Figure 1A, right panel and Figure 1B). In healthy donors, the baseline level of CD25 expression was sometimes higher (Figure 1B) compared to HCV patients, but there were no major differences between baseline and HCV pp stimulation. Consistent with the manufacturer's technical datasheet, freshly isolated cells expressed more homogenous and intermediate levels of CD25 (Figure S1). These data supported our previous observation with core and NS5 peptides [14], that a proportion of natural CD25^+^ cells can sustain and/or up-regulate CD25 expression (now termed CD25^+/↑^ cells) in the presence of HCV peptides and this phenomenon is likely to be disease specific.

### Epigenetic modification of FOXP3 locus in HCV-responsive natural CD25^+^ cells

The transcription factor FOXP3 plays a critical role in the development and function of natural Treg, but in humans this molecule is also transiently expressed by activated conventional T cells [15],[16]. We have recently shown that epigenetic DNA modification of an evolutionarily conserved element within the *FOXP3* locus, named Treg-specific demethylated region (TSDR), correlates with a stable Treg phenotype [17]. In the current study, we applied this principle to determine whether the CD25^+/↑^ cells, which were previously shown to express FOXP3 [14], are Treg or activated conventional T cells.

**Figure 2:**
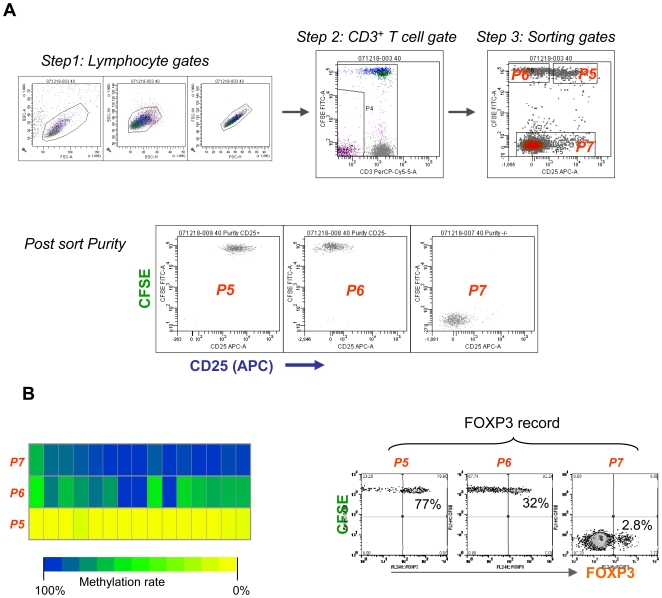
Methylation analysis of the FoxP3 locus in sorted cell populations. (A) Gates for sorting and post-sort purity. The co-cultured cells from a patient were sorted at day 5, based on CD25 expression, into 3 fractions: P5 (CFSE^+^CD25^+^), P6 (CFSE^+^CD25^−^) and P7 (conventional T cells, CD3^+^CFSE^−^) using BD FACSAria. Note that a CD3 gate was introduced to the CFSE^−^ fraction in addition to the lymphocyte gate. This sorting strategy was applied to both methylation (N = 5) and microarray (N = 6) experiments. (B) Methylation analysis. The left panel shows the methylation status of the TSDR in the sorted populations P5, P6 and P7. Each block represents a CpG motif of the TSDR (Amp5) within the FOXP3 locus. The methylation level is colour coded (yellow = 0% methylation, blue = 100% methylation). The panel on the right shows the FOXP3 record of sorted cell fractions for this patient. (Representative data of N = 5 patients).

HCVpp stimulated CFSE-CD25^+^/CD25^−^ co-cultures were FACS sorted on day 5 into 3 fractions (Figure 2A): CD25^+/↑^ cells (P5, >95% of which are CD4^+^, Figure S2), CD25^low^ (P6) and conventional T cells (P7). Analysis of DNA purified from the above sorted cells by bisulphate sequencing revealed (Figure 2B, left) a highly demethylated TSDR in the HCV-responsive fraction (CD25^+/↑^ cells, P5), which suggest that these cells are true Treg with stable FOXP3 expression and function. As expected, the TSDR in the conventional T cell fraction (P7) remained highly methylated. The TSDR in the HCV-non-responsive fraction (CD25^low^ cells, P6) showed various degrees of demethylation, which reflects a mixed population of known or unknown cell types. Some P6 cells expressed FOXP3 (Figure 2B, right), but the proportion varied greatly among patients (from ∼5% to ∼40%, data not shown).

### Global gene expression profiling for HCV-responsive Treg

**Figure 3:**
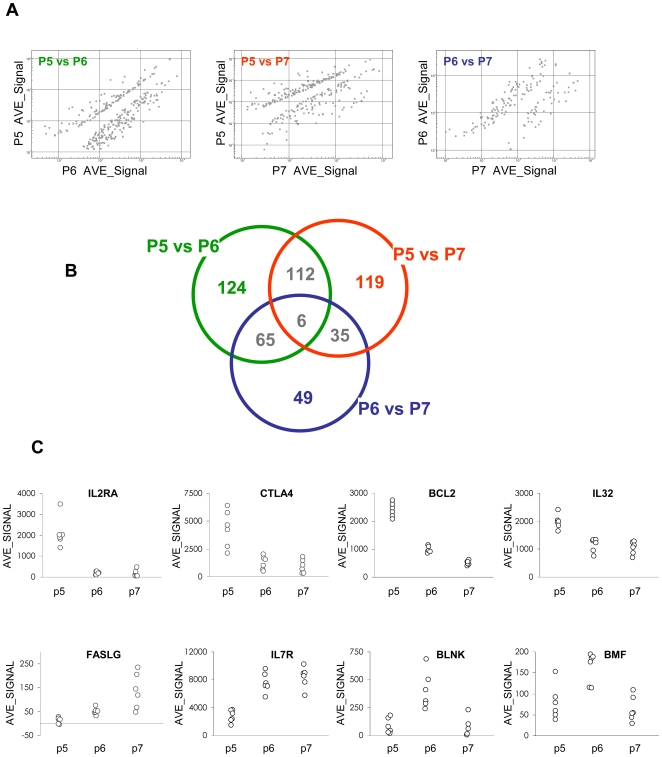
Microarray data. (A) The overall pattern of transcript changes in dot plots; each dot represents a gene. The cut-offs used were: Diff Pval <0.05 and fold change >1.5. (B) Venn diagram showing the number of genes that were differentially expressed between P5 and P6, P5 and P7, and P6 and P7. Note that the transcription levels of certain genes are changed in more than one comparison. (C) Typical examples of differentially expressed genes, each data point represents the average value of all technical replicates (background subtracted and normalized) of one patient. The data shown in (A) and (B) were derived from statistical calculations based on a sample size of N = 6, whereas the data shown in (C) represent the data generated from individual patients.

To further understand the putative disease-associated CD25^+/↑^ Treg, genome-wide transcriptional profiles were generated on RNA isolated from the cells, cultured and sorted as described above (Microarray datasets are deposited in Gene Expression Omnibus under series record GSE16576, and can be reviewed via the following link: http://www.ncbi.nlm.nih.gov/geo/query/acc.cgi?acc=GSE16576). The Illumina platform was chosen because it requires only 100ng RNA, and given that cell numbers in P5 (CD25^+/↑^) and P6 (CD25^low^, or HCV-non-responsive natural CD25^+^ cells) were limited, this allowed us to analyse each patient individually without pooling samples and thus permit rigorous statistical analysis. Of ∼46,000 genes (or probe sets) analysed, 307 genes were differentially expressed between P5 and P6, followed by 272 genes differentially expressed between P5 and P7 and 155 genes between P6 and P7 (Figure 3A). Some transcript changes were found in more than one comparison (Figure 3B). This constitutes ∼1% of the entire known transcriptome, while the remaining ∼99% of genes were expressed at similar levels by all three T cell fractions. Table S1 provides the full list of genes that were differentially expressed in P5 compared to P6 or P7 (Table S1-A), and in P6 compared to P7 (Table S1-A). Figure 3C shows selected examples of these genes and demonstrates that the data are highly reproducible.

The key Treg signature genes, such as FOXP3, GITR, CD25, IL7R and CTLA4 were differentially expressed as expected among the 3 fractions (Table 1 and Figure 3C) and provide confidence that the experimental system was able to generate quality data. A number of transcription factors (Table 1 and Table S1) were among the differentially expressed genes. This is not particularly surprising because studies in mice suggested that transcription factors are among the genes regulating or regulated by Foxp3 [18].

**Table 1:** Examples of differentially expressed genes in P5 compared to P6 and/or P7.

Group, gene symbol	AVG_SIGNAL			P5/P6 Log_2_FC	Diff Pval	P5/P7 Log_2_FC	Diff Pval
	P5	P6	P7				
*Treg markers or associated*							
CD127	2767	7597	8349	−1.457	1.9E-08	−1.593	3.3E-07
CD40L	148	308	291	−1.054	2.1E-03	−0.974	7.9E-03
CTLA4	4317	1244	939	1.795	5.0E-03	2.201	1.3E-03
FOXP3	84	22	19	1.904	3.7E-07	2.172	9.7E-08
GITR	226	36	42	2.652	2.7E-02		
GJB6	85	12	7	2.870	2.8E-03	3.535	1.3E-03
IL2RA	2104	181	155	3.541	4.0E-08	3.767	8.4E-08
IL2RB	5469	2362	3580	1.211	8.3E-06		
*Apoptosis*							
BCL2	2400	984	505	1.287	5.7E-08	2.249	1.6E-16
BCL2L1	657	275	351	1.253	7.7E-05	0.905	9.4E-03
BMF	79	162	63	−1.038	4.3E-02		
CD120b	4965	2177	1882	1.190	4.3E-07	1.399	1.3E-07
*Cytokines*							
IL7	42	36	1			5.034	1.0E-02
IL15	87	280	189	−1.678	0.001		
IL32	1984	1149	1044	0.788	3.6E-03	0.926	6.6E-04
*Killing*							
FASLG	13	51	137	−1.989	2.7E-03	-3.403	1.5E-02
GZMA	325	717	4537			-3.802	2.6E-34
GZMK	258	655	3450			-3.743	2.6E-34
KLRD1	29	96	819	−1.709	1.3E-02	-4.804	1.4E-07
KLRK1	45	203	1378	−2.172	2.2E-03	-4.937	2.6E-34
PRF1	836	488	2574	0.775	1.3E-02		
*Transcription factors*							
CITED4	618	194	214	1.672	2.8E-04	1.527	7.0E-04
E2F5	214	555	180	−1.372	9.1E-03		
PCAF	2698	1710	1607	0.658	3.0E-02	0.748	1.4E-02
PFTK1	71	248	57	−1.806	1.6E-02		
UBTF	144	188	250			−0.798	4.7E-02
ZNFN1A4	685	108	69	2.668	7.1E-17	3.317	1.7E-20
*Cell cycle, proliferation*							
FLT3LG	863	384	392	1.168	2.5E-04	1.140	7.9E-04
TPD52L1	51	15	9	1.747	4.2E-04	2.520	6.9E-06
*B cell function associated*							
BLNK	84	405	67	−2.265	3.3E-03		
CD19	389	2026	405	−2.379	1.4E-08		
CD72	161	656	242	−2.023	5.2E-03		
FCRL1	84	420	86	−2.329	2.5E-12		
FCRLM1	473	2371	447	−2.327	1.8E-06		
*Others*							
ATP1B1	320	149	108	1.101	3.3E-02	1.574	1.8E-03
CCL5 (RANTES)	116	639	3487	−2.465	1.7E-06	−4.913	3.9E-09
CCR7	3351	4220	6376			−0.928	6.6E-03
CD161	694	1771	1633	−1.351	1.7E-02		
CISH	2649	399	247	2.730	3.1E-15	3.423	3.0E-18
CLAUDIN2	18	28	58			−1.660	3.1E-02
CXCR7	258	137	120	0.913	1.5E-03	1.103	3.0E-04
GNG2	583	354	339	0.722	6.1E-02	0.783	4.1E-02
IFNAR2	1199	844	721			0.735	2.0E-02
IL18RAP	349	349	1291			−1.887	1.0E-04
TLR6	28	80	25	−1.514	1.7E-03		
TLR9	55	107	48	−0.961	1.1E-02		
TLR10	98	574	95	−2.554	1.4E-12		
IRF4	127	81	45			1.492	4.9E-04
IRF8	716	2410	927	−1.751	2.2E-04		
F2R (thrombin receptior)	14	48	148	−1.822	7.8E-04	−3.440	1.7E-04
PDL1	157	92	60			1.390	6.6E-05
PTGER2 (PGE2)	935	441	254	1.882	2.8E-03		
SOCS2	746	129	48	2.527	3.2E-02	3.956	6.7E-03
TGFBR2	836	1264	1499			−0.843	2.6E-02
TGFBR3	332	592	999			−1.592	1.5E-02

Footnotes: The AVE_SIGNAL is background subtracted hybridization fluorescent intensity (see materials and methods), and all genes have a detection P value<0.001 for P5 (data not shown). For differential analysis, the cut off was set at diff Pval <0.05 and fold change >1.5 (Log_2_FC>0.58 or <−0.58), and the data space was left empty when this criterion was not met. (Please see Table S1 for the full list of differentially expressed genes).

Ingenuity Pathway Analysis (Ingenuity Systems, www.ingenuity.com), a literature based online annotation tool, was used to identify the relationships and biological significance of the affected genes (Figure S3 and S4). This is the first study in which the putative HCV-specific Treg (CD25^+/↑^) were analysed against the putative non-HCV-specific Treg (P6), as well as conventional T cells (P7). Most interestingly, a group of genes (Table 1, Figure 3C and Table S1) that were known to be implicated in T cell survival or proliferation (within the top function, immune response, in Figure S3) were differentially expressed by P5 compared to P6 and/or P7. This includes the up-regulation of BCL2 and BCL2L1 (anti-apoptosis), TNFRSF1B and FLT3LG (promote T cell proliferation and activation), IL7 (T cell survival signal) and IL32 (a cytokine released following T cell activation, reviewed in reference [19]), and the down-regulation of the pro-apoptosis gene BMF. This pattern suggests that cells in P5 are likely to be more activated and perhaps have a survival advantage over cells in P7 and/or P6. Figure S3 summarizes the major networks of interactions between these affected genes.

**Figure 4:**
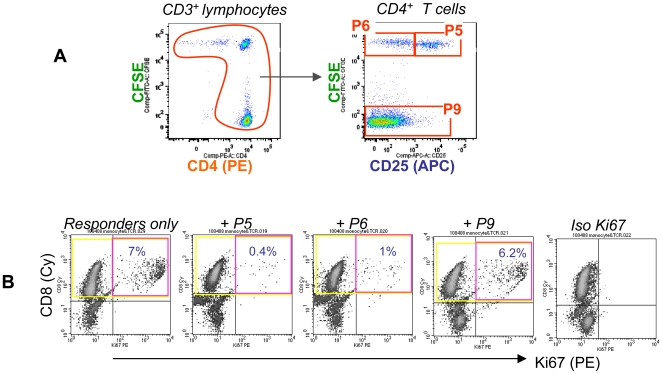
CD25^+/↑^ cells are suppressive. (A) A simple illustration of the CD4 gate. The co-cultured cells from the patient were sorted as described in Figure 2A, except that a CD4 gate was introduced in addition to the CD3 gate, into P5 (CFSE^+^CD25^+^), P6 (CFSE^+^CD25^−^) and P9 (CD4^+^CD3^+^CFSE^−^) fractions. (B) The *in vitro* suppression assay. The target cells were a HCV-specific CD8 T cell line (comprised of autologous CD8 T cells and monocytes, cultured for 5 days in the presence of HCVpp). 1×10^5^ of the sorted cells (P5, P6 or P9) were mixed with 2×10^5^ target cells and 2×10^4^ feeders (autologous iDC). This co-culture was stimulated with HCVpp for 7 days, and analysed for Ki67 expression by flow cyometry, gating on CD8^+^ target cells. (Representative data of N = 3 independent experiments using different donors).

It is known (reviewed in [11]) that Treg must be activated via their TCR to gain suppressor function, and we applied this principle to test the activation status of CD25^+/↑^ cells (N = 3). We used CD4^+^ conventional T cells as control because the CD25^+^ cells isolated from PBMC were almost exclusively CD4^+^ (Figure S2). The responder cells were a short term autologous CD8^+^ T cell line driven by HCVpp. The sorted cells (see Figure 4A for a simple illustration and Figure 2A for technical details) were added to responder cells at a ratio of 1∶2 and cultured for 7 days. CD25^+/↑^ cells strongly suppressed HCV-specific CD8^+^ T cell proliferation, as measured by Ki67 expression on the responder cells (as the effector frequency is low in HCV patients we found that the Ki67 assay is more sensitive than 3HTdR incorporation in assays with low proliferating cell numbers). Cells from P6 suppressed to a lesser degree, reflecting that this was a mixed population of various cells of unknown nature, while conventional CD4^+^ cells had no suppressive activity (Figure 4B). These results were confirmed in studies with cells from two additional patients (data not shown). In addition to suppression, P5 also expressed a higher level of IL32 mRNA than P6 (Table 2, in 3 of 4 patients) and P7 (Table 2, in 4 of 4 patients), analysed by qRT-PCR. The role of IL32 in HCV infection is unknown and requires future investigation. Taken together, P5 at the population level correlated with cytokine production and suppressor function, although at present we do not have a reporter molecule that could independently validate the TCR recognition of HCV antigens at the single cell level, a challenging area that is currently being investigated in our laboratories.

**Table 2:** Real time RT-PCR detection of IL32 in N = 4 chronic HCV patients.

Patient ID	Fraction	IL32 relative copy numbers
	P5	4.814
PH12	P6	1.153
	P7	2.66
	P5	7.159
SA76	P6	10.364
	P7	1.503
	P5	5.225
S07-37	P6	0.807
	P7	1.281
	P5	33.757
S07-40	P6	4.240
	P7	1.290

A number of genes related to B cell phenotype and function, such as toll like receptors, CD19, CD72, CD86, BLNK, etc. were up-regulated in P6. Interestingly, the same category of genes was also up-regulated in healthy donor natural Treg compared to conventional T cells (Barry, unpublished data). The implication of this is currently unclear. Genes related to CD8^+^ T effector cell functions (such as CD8, perforin and granzymes) were upregulated in P7 (Table 1 and Table S1), consistent with the fact that this was the only fraction which contained CD8^+^ T cells, while the original CD25^+^ fraction (now P5 and P6) contained mainly CD4^+^ cells (Figure S2).

### Identity of putative Treg epitopes

The HCV NS3 protein has been proposed as a suitable immunogen for vaccine development [20]. The NS3 peptide array (provided by BEI resources, ATCC) consists of 97 overlapping peptides that cover the length of this protein (Table S2 lists the sequence of each peptide). We tested each of the peptides for their ability to induce CD25^+/↑^ cells following individual peptide stimulation (N = 8). Our working hypothesis is that such a phenomenon directly or indirectly reflects Treg recognition of HCV antigens.

**Figure 5:**
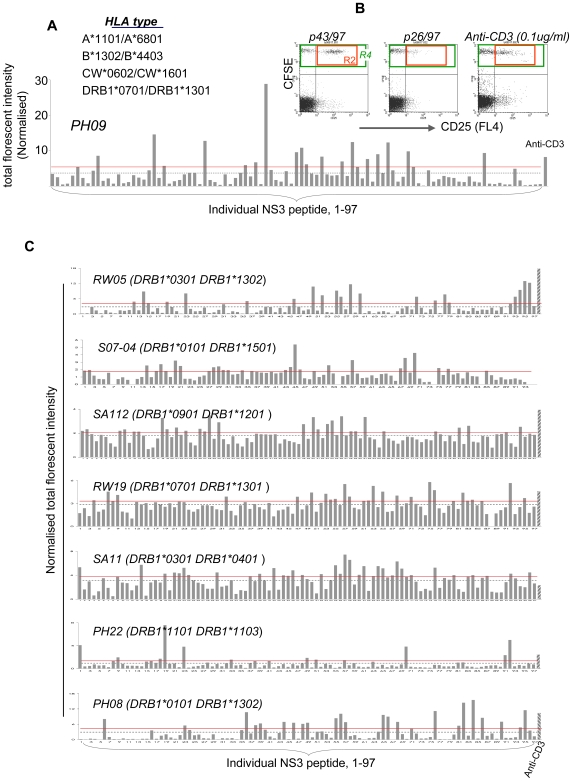
Defining the target antigens for HCV-specific Tregs. (A) The CFSE-CD25^+^/CD25^−^ co-culture from one patient was stimulated in a 96-well plate with individual NS3 peptides, genotype matched, and analysed for CD25 expression on day 5. The bar plot shows total fluorescent intensity (TFI = % x MFI) of CD25^+^ cells (R2 within R4) induced by each peptide for this patient. TFI was normalised against the MFI of CFSE^+^/CD25^−^ population (upper left quadrant). The dotted line represents the average TFI of all peptides tested and the solid line indicates the cut off (average + 4 standard errors). (B) Typical plots of reactive and non-reactive peptides. The plot p43/97 depicts the typical profile by a few reactive peptides, whereas p26/97 depicts the lack of response by a majority of the peptides. The positive control was anti-CD3 (0.1 µg/µl final). (C) NS3 mapping data from an additional 7 patients. PH08 spontaneously resolved the infection and the remainder are chronically infected. S07-04 is genotype 1a and the remainder are genotype 3a.

Comprehensive HLA typing of all common loci including class I (HLA-A, B, C) and class II (HLADRB1) was performed for each patient by DNA-based sequencing methods (Figure 5 and Table S3). We found, as expected, that the HLA diversity amongst individuals was high, which may explain why the reactive peptides were not overtly consistent among patients. While the exact location varied among patients, for a given patient, only a few peptides could induce CD25 up-regulation (Figure 5), which is consistent with our earlier findings with the HCV core protein [14]. Some of the reactive peptides are located close to or overlapped with previously described T cell epitopes (Table S4). The implications of this need to be further investigated.

**Figure 6:**
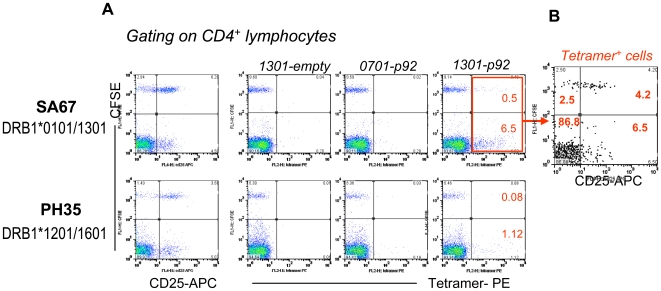
Tetramer staining. (A) The HCV peptide pool stimulated CFSE-CD25^+^/CD25^−^ co-culture was harvested on day 5 and stained with the test tetramer 1301-p92, or control tetramer 1301-empty and 1701-p92. SA67 is a HLA matched donor (DRB1*0101/DRB1*1301) and PH 35 is a HLA mismatched control (DRB1*1201/DRB1*1601), both chronically infected with HCV gt3a. The numbers indicate the percentage of CD4^+^ lymphocytes within the quadrant. (B) CD25 expression profile of tetramer^+^ cells. The numbers indicate the percentage of tetramer^+^ cells within the quadrant.

The mechanisms of the positive responses are unknown but our data suggested that it could be related to the HCV-specific nature of Treg. To test this working hypothesis, we designed a HLA (DRB1*1301)-peptide (WKCLVRLKPTLHGPTPLL, the p92) tetramer, which is, to our knowledge, the only HCV HLA class II -peptide tetramer developed based on non-T-helper responses. Compared to a HLA mismatched control, more tetramer^+^ cells were detected in the patient with DRB1*1301 (7% in SA67 compared to 1.2% in PH 35 in Figure 6A), suggesting the staining signal is likely true. The control tetramers 0701-p92 (mismatched HLA loaded with the same peptide) and 1301-empty (the correct HLA but loaded with no peptide) showed minimal background staining, further suggesting that the staining is genuine. Importantly (Figure 6B), a high proportion (>60%) of the tetramer^+^ Treg cells were CD25^+^, while the vast majority (>90%) of tetramer^+^ T-helper cells were CD25^−^, supporting our hypothesis and also implying that the tetramer^+^ T-helper are likely not functional (given that CD25 is an activation marker for conventional T cells).

## Discussion

Conventional protocols to culture human Treg usually involve long term expansion in the presence of high doses of rhIL2. We have previously described a novel co-culture system [14], which we believe to be more physiological. In this system, PBMC-derived CD25^+^ cells are labelled with CFSE, mixed with CD25^−^ cells from the same donor and finally stimulated with HCV peptides. This approach, used throughout the current study, allowed us to identify a HCV-specific response within the natural CD25^+^ cell population by observing their response to HCV antigen with conventional T cells as an internal control. We found that the CD25^+^ population isolated from PBMC of HCV patients, despite a failure to proliferate (which is consistent with the literature that Treg are hypo-proliferative *in vitro*), responded to HCV peptide stimulation by sustaining and/or up-regulating CD25 surface expression, a phenomenon that does not occur, or at least to a lesser degree, in healthy donors. It is not known if human Treg can down regulate CD25 expression *in vitro* in the absence of antigen, but we think this can not be excluded.

In naïve inbred pathogen-free mice, CD25^+^ cells isolated from PBL are almost entirely Foxp3^+^ natural Treg, but in adult humans, the CD25 expression level is more heterogeneous, as this population is expected to contain activated effector T cells and other known or unknown cell types, particularly during infection. The transient expression of FOXP3 by activated human conventional T cells [15],[16] further complicates the interpretation of human data. We found that natural Treg and Treg converted *in vivo* under tolerogenic conditions [21] exhibited a completely demethylated TSDR, whereas activated conventional T cells and TGF-β induced Treg contained almost 100% methylated CpG motifs. We therefore proposed the TSDR methylation status as a reliable criterion for the identification of natural and stable subsets of induced Tregs [17]. Using the same criteria, we confirm here that the CD25^+/↑^ cells in our culture are not activated conventional T cells or TGF-β converted unstable Treg, but are “hard-wired” stable Treg. Since the origin of human Treg is unclear [22],[23], CD25^+/↑^ cells could either belong to the natural Treg lineage, or be converted from peripheral HCV-specific conventional T cells during the infection, but if it is conversion, the conversion is thorough, as demonstrated by the epigenetic imprint. More Treg were found in HCV-infected liver than periphery blood [24], where a surprisingly high proportion (∼80%) of T cells expressed FOXP3. *In vivo* expansion of HCV-specific Treg is possible, as Treg from a HCV-experienced chimpanzee had a lower TCR excision circle content compared to naïve animals [25]. The induction and expansion of HCV-specific Treg could have profound effects on the quantity and quality of the anti-viral effector T cell responses.

We next generated gene expression profiles of CD25^+/↑^ cells (P5), using CD25^low^ (P6) and conventional T cells (P7) as controls, to understand the molecular program that governs the role of these cells. In addition to typical Treg gene patterns, which are either consistent with our FACS data or with the literature, P5 also expressed genes patterns that are less known, such as the survival profile. In an independent study (Barry, et al, unpublished data) we generated transcriptional profiles for *ex vivo* isolated (FACS sorted CD25^high^ cells) resting, as well as polyclonal stimulated Treg and conventional T cells from healthy donors. Comparing our current dataset to the healthy donor dataset provides hints as to transcriptional changes which could be unique in HCV patients and thus likely to be associated with HCV infection. BCL2, BMF, IL7, IL32, CISH, CCL5, CCR7, IFNαR2, IRF4 and IRF8 (Table 1 and Table S1) are all among this “unique” list, and these genes are known to be critical in regulating cell survival or play important roles in immune responses against pathogens. Development of these data is necessary and is currently ongoing in our laboratories. It was recently reported that the gene profile of *ex vivo* isolated total Treg from HCV patients was very similar to that of healthy donors [26], as only 5 genes were differentially expressed between the two and the change ranged from 0.4 to 2. Interestingly, none of these 5 genes was identified in our experiments. We think that Treg and non-Treg compartments are both likely to be affected by the disease, a detail which would not be revealed by comparing total Treg of patients and healthy donors.

The continued expression and/or up-regulation of CD25 on a proportion of Treg in response to HCV peptide stimulation *in vitro* is an event associated with HCV infection, because it does not occur, or is greatly reduced, in healthy donors. This could be a consequence of TCR engagement by the HCV antigen in the context of the peptide/HLA complex, a view supported by the suppression assay data, or alternatively, IL2 (and/or other soluble factors) produced by effector T cells within the co-culture may affect CD25 expression on Treg independently of antigen recognition. In the latter scenario, the apparent antigen specificity of Treg is likely to reflect the antigen specificity of the effector T cells. However, the effector frequency within PBMC was very low, as suggested by the literature (reviewed in reference 2). Supernatant IL10 and IFN-γ levels (measured using Cytokine Bead Array, BD Biosciences) also did not consistently correlate with culture conditions viz. the CFSE-CD25^+^/CD25^−^ co-cultures and the CD25^−^ PBMC cultures with or without antigen, from patients or from healthy donors (data not shown), and IL2 was generally below the detection limit (data not shown). This is consistent with our microarray data, as none of the key gene signatures for Th1/Th2, Th3 and Th17 (IL2, IL4, IL10, IFNγ, IL12p70, IL17, TGFβ, IL6, etc.) were differentially expressed among the fractions upon HCV antigen stimulation. Thus it is unlikely that the common soluble factors produced by conventional T cells or other antigen non-specific cells in culture could determine the apparent Treg responsiveness.

Ideally we should use a Treg-specific activation marker for epitope mapping, but since there is no such marker we used CD25 as a surrogate marker. In almost every patient, the most reactive NS3 peptide induced higher CD25 expression on Treg compared to anti-CD3 (Figure 5B). Given that anti-CD3 induced more conventional T cells to express CD25 than any of the peptides (Figure 5B and data not shown), these data support the concept that soluble factors alone do not completely correlate with the magnitude of the Treg response, as the level of IL2 in the anti-CD3 culture must be otherwise sufficient to achieve the highest CD25 expression. We attempted to match the reactive peptides against published data on effector T cell epitopes, but found this difficult, as studies using class II tetramers only focus on a few epitope/DR pairs, while in studies which did not use tetramers the HLA typing data were incomplete or missing. Further validation of the putative Treg epitopes and their HLA restriction are required, but nevertheless, our data show that the breadth of the reactivity is rather narrow, while the response itself is robust.

Due to the lack of any Treg specific surface marker and a simple functional readout for these cells, it has not been possible to develop tetramers that are restricted to Treg. Using two class II HLA tetramers previously developed based on T-helper responses, Heeg et al [27] detected FOXP3^+^ cells during acute infection and reported that the frequency of tetramer^+^FOXP3^+^ cells was low and did not correlate with disease progress or outcome. It is unclear at present how this reflects a global picture of Treg/Teff balance, as it is not known to what extent the Treg repertoire overlaps with that of Teff, or if Treg and Teff clones of the same antigen specificity would expand/contact with the same kinetics. Unfortunately, our tetramer data is limited at present and could not answer these questions. Further studies are required, but since it is impossible to develop tetramer for every T cell epitope, we believe that it is important to develop a higher throughput or a more practical Treg antigen specificity readout so that a more global picture can be obtained.

This study opens a window to explore the role of Treg and their target antigens in a chronic viral infection of humans. The target antigens recognised by the FOXP3^+^ lineage in humans are largely unknown and systems to guide the discovery of these antigens would benefit future studies in HCV vaccines and immunotherapy.

## Materials and Methods

### Ethics statement

The study was approved by the Alfred Hospital Ethics Committee and the Victorian Department of Human Services Human Research Ethics Committee. Written informed consent was obtained from each subject.

### Subjects

HCV-infected participants (N = 31) were recruited from the Alfred Hospital, Melbourne and from an ongoing study of hepatitis C virus in the social networks of injecting drug users. All participants were HCV mono-infected, with either genotype 1a or genotype 3a viruses, and one participant resolved the infection spontaneously. A few patients were treated previously (unsuccessfully) with interferon/ribavirin and the remainder were untreated. Healthy donors were represented by local volunteers or blood donors from the Australian Red Cross Blood Transfusion Service, Melbourne Branch.

### Antigens

The HCV peptide array, which contains 18-mer peptides overlapping by 11aa covering the entire HCV polyprotein, for genotype 1a and 3a were provided by BEI Resources, ATCC. A peptide pool (pp) working stock (containing 100 µg/ml of each peptide) was prepared in DMSO/RPMI. The final concentration of HCVpp within the culture was 0.2 µg/ml in initial experiments and 0.15 µg/ml for subsequent experiments, or as indicated.

### Cell culture

PBMC from patients or healthy donor controls were separated by Ficoll Paque centrifugation and CD25^+^ cells were isolated from PBMC using CD25 microbeads (MiltenyiBiotec) according to the manufacturer's instructions. The CD25^+^ cells, typically 1–2% of total PBMC, were labelled with CFSE (Sigma-Aldrich) and mixed back with unlabeled CD25-depleted PBMC at a ratio 1∶10. The CFSE-CD25^+^/CD25^−^ co-culture was stimulated with or without genotype matched HCVpp in RPMI-1640, 2 mM L-glutamine, 100 IU/mL penicillin-streptomycin (Invitrogen) and 5% human AB serum (MP Biosciences) in 24-well tissue culture plates (Interpath, Australia). Cells were harvested on day 5 for flow cytometry analysis or sorting. In some experiments, culture supernatants were collected prior to cell harvesting for cytokine analysis at later stage.

### Antibodies and flow cytometric analyses

In general, fluorescent dye-conjugated antibodies and isotype controls were purchased from BD Biosciences. PE-conjugated anti-human FOXP3, isotype control and FOXP3 staining buffer set were purchased from eBiosciences. Intra-nuclear staining of FOXP3, as well as Ki67, was performed according to the manufacturer's instructions.

Flow cytometry was performed using a FACScalibur flow cytometer (BD Biosciences,) and Cellquest software. For data analyses, an initial lymphocyte gate was set based on SSC/FSC and additional gates introduced as required. Results are presented as the percentage, or mean fluorescent intensity (MFI) of positively stained cells within the gated population.

### Sorting

Sorting of HCV peptide-stimulated CFSE-CD25^+^/CD25^−^ co-cultures from HCV patients was performed using a FACSaria located in a PC3 facility. The cultures were sorted on day 5 into 3 fractions as specified, based on their CFSE labelling and CD25 expression. The primary gate was set on lymphocytes based on SSC/FSC and an additional CD3 gate (for methylation analysis and microarray) or CD4 gate (for *in vitro* suppression assay) was introduced to the CFSE^-^ population to refine the conventional T cell population.

### FOXP3 DNA methylation analysis

For this series of experiments, we used cells from male patients, as this overcomes the potential X-chromosomal inactivation of one *FOXP3* allele, which usually affects the methylation analysis of Treg in females. Genomic DNA was isolated from sorted cells (Figure 2A) using NucleoSpinTissue XS kit (Macherey & Nagel, Düren, Germany) following the protocol for cultured cells. Bisulfite treatment of genomic DNA was performed as described previously [28] TSDR-primers (5′ to 3′ direction) p-TGTTTGGGGGTAGAGGATTT and o-TATCACCCCACCTAAACCAA, amplifying Amp5 [17] were used for bisulphite-specific PCR and sequencing reactions. The primers “p” and “o” produce amplicons based on the +1 strand.

PCR was performed in a final volume of 25 µl containing 1x PCR Buffer, 1U *Taq* DNA polymerase (Qiagen), 200 µM dNTPs, 12.5pmol each of forward and reverse primers, and 7ng of bisulphite-treated genomic DNA at 95°C for 15 min and 40 cycles of 95°C for 1 min, 55°C for 45 sec and 72°C for 1 min with a final extension step of 10 min at 72°C. PCR products were purified using ExoSAP-IT (USB Corp.) and sequenced using the PCR primers and the ABI Big Dye Terminator v1.1-chemistry (Applied Biosystems) followed by capillary electrophoresis on an ABI 3100 genetic analyzer. AB1 files were interpreted using ESME.

### RNA purification and microarray analysis

Total RNA from sorted cells (P5 = CD25+CFSE+, P6 = CD25−CFSE+ and P7 = CD3+CFSE−, as illustrated in Figure 2A) was isolated using RNeasy Kit (QIAGEN Australia) according to the manufacturer's instructions. The RNA quality was ascertained by the Agilent Bioanalyser 2100 using the NanoChip protocol.

The microarray experiments were performed, according to the technical manual from Illumina, by the Australia Genome Research Facility. In brief, 100 ng RNA was amplified using the Illumina Total Prep RNA amplification kit (Ambion Cat. No. IL1791) to generate biotinylated cRNA. An aliquot (1.5 µg/30µl) of the labeled cRNA for each sample, prepared in a probe cocktail that included GEX-HYB Hybridization Buffer, was hybridized to an Illumina Sentrix Human-6 Expression BeadChip-v2.0 at 58°C for 16 hours. After hybridization, the chips were washed, coupled with streptavadin-Cy3 and scanned in the Illumina BeadArray Reader. The scanner operating software, BeadStudio, converts the signal on the array into a TXT file for downstream analysis.

### Microarray data analysis

Data analysis and visualization were performed using BeadStudio Gene Expression Module v3.3 software (Illumina Inc., San Diego, CA). With Illumina gene expression array, each probe is measured at least 30 times independently on random distributed beads. This large number of technical replicates allows robust estimation of the hybridization intensity and the measurement error for each probe. The signal for each probe or probe set (gene) was averaged and the background (the average signal from the large number of randomly distributed negative control beads) subtracted, and then normalized using quantile algorithms that account for variations between probes and between chips. A detection P value, calculated by comparing the distribution of the transcript signal to that of the negative control signal, was set at ≤0.001 to identify transcripts that were expressed (with a confidence of ≥99.9%) above background. Genes with detection P value≤0.001 in at least one of the three fractions were selected for further analysis. To detect changes in gene expression between samples, the differential P value (Diff Pval) was calculated using the Illumina custom error model, which allows 5% false discovery rate being automatically adjusted. The cut off for the Diff Pval was set at ≤0.05 (a confidence of ≥95% that the given gene is expressed at different levels between the sample and control).

We used the Ingenuity Pathway Analysis online software (Ingenuity Systems, www.ingenuity.com) to help further group the genes in term of networks and functions.

### Real time RT-PCR

RNA was isolated from sorted cells as above. Real time RT-PCR assay was performed using Mx3000P QPCR system (Agilent Technologies). The gene expression assays for IL32 and house keep control GAPDH, as well as One-Step Master Mix Reagents, were purchased from Applied Biosystems (Foster City, CA, USA). The cycle conditions are 30 min at 48°C for cDNA synthesis, 10 min at 95°C, followed by 50 cycles of 15 sec 95°C, 60°C 1 min. Data were analysed using MxPro software supplied by the manufacturer.

### In vitro suppression assay

The co-culture was sorted by FACSAria to CD25^+^CFSE^+^ (hypothetical HCV-specific Treg), CD25^−^CFSE^+^ (Treg of other specificity and other unavoidable contaminating cells) and CD4^+^CFSE^−^ (conventional CD4^+^) in a PC3 facility. The target cells were represented by an autologous HCV-specific CD8^+^ T cell line, for which an equal number of CD8^+^ T cells and CD14^+^ monocytes were mixed and cultured in the presence of 0.15 µg/ml HCVpp for 5 days. The *in vitro* assay was set up in U-bottom 96-well plates in triplicate. Each well, in a final volume of 200 µl, contained 1×10^5^ sorted cells, 2×10^5^ target cells and 2×10^4^ feeder (autologous immature dendritic cells generated as described previously [29]) and the antigens HCVpp (0.1 ug/ml final of each peptide). At the end of the culture period (day 7), cells were pooled from the triplicate wells, stained for Ki67 expression and analysed by flow cytometry, gating on CD8^+^ lymphocytes (note that the sorted cells in this experiment were CD4^+^).

### NS3 Treg epitope mapping

The CFSE-CD25^+^/CD25^−^ co-cultures were set up essentially as described above, except in a 96 well format, containing 2×10^5^cells in 200 ul medium. Each individual NS3 peptide (Table S1), genotype-matched, was added to each different well at 10 µg/ml final. Anti-CD3 (clone 32-2A2, Mabtech) was used as a positive control at 0.1 µg/ml final. The cultures were harvested on day 5 and analysed for CD25 expression by flow cytometry. The criteria for reactive peptides were described previously [14].

### Tetramer staining

The p92, WKCLVRLKPTLHGPTPLL, is located towards the C terminal of NS3 of HCV genotype 3a (Table S1). PE conjugated HLA class II-peptide tetramer complexes (DRB1*1301-p92, DRB1*0701-p92 and DRB1*1301-empty) were synthesized at the Benaroya Research Institute, USA. For staining, the CFSE-CD25^+^/CD25^−^ co-culture was harvested at day 5, washed and resuspended in fresh RPMI medium (same as for culture but without HCV peptides) at 1×10^5^ cells in 50 ul per well. To each well 1 ul of a tetramer was added and the cells incubated for 3 h at 37°C, then 30 min at 4°C to stain surface molecules CD25 and CD4.

### HLA genotyping

High-resolution HLA Class I and II typing was performed by direct DNA sequencing methods as previously described [30]. Ambiguities were resolved following sequencing with allele-specific subtyping primers. Sequence electropherograms were analysed using Assign™ (Conexio Genomics). Allele assignment was based upon identity at exons 2 and 3 and consistently allocated for the most common expressed allele in the relevant population.

## Supporting Information

Table S1 Full list of differentially expressed genes
(0.35 MB PDF)

Table S2 NS3 peptide array used in the current study
(0.04 MB PDF)

Table S3 HLA typing data
(0.07 MB PDF)

Table S4 NS3 T cell epitopes found in the literature.
(0.08 MB PDF)

Figure S1 CD25 and FOXP3 expression profile of freshly isolated CD25^+^ cells. Freshly isolated CD25^+^ cells were stained for CD25 (surface) and FOXP3 (intracellular), followed by flow cytometry analysis gating on CD3^+^ lymphocytes (representative data from N = 3 HCV patients).
(0.05 MB PDF)

Figure S2 CD4 expression on day 5 of the co-culture. Plot (A) shows the lymphocyte gate, (B) shows the CD25 expression on the lymphocytes, (C) depicts CD4/CD3 expression on CFSE^+^CD25^+^ population (corresponding to P5, HCV-responsive Treg), (D) CD4/CD3 expression on CFSE-CD25^+^ population (corresponding to P6, HCV-non-responsive Treg) and (E) shows CD4/CD3 expression on CFSE unlabeled CD25 depleted fraction, the CD3^+^ cells are conventional T cells, corresponding to P7. (Representative data from N = 5 HCV patients).
(0.08 MB PDF)

Figure S3 Ontology clustering using Ingenuity Pathway Analysis. The differentially expressed genes between P5 and P7 (dark blue) and between P5 and P6 (light blue bar) were grouped into 9 major functional clusters (x-axis). The y-axis indicates the Fisher's exact test P-value, that the higher the bar the less likely the genes might be found together owning to chance alone.
(0.09 MB PDF)

Figure S4 Network view illustrating relationships between differentially expressed genes. To construct networks, IPA overlays the differentially expressed genes with a literature based global molecular network, and identifies connections between these genes. A total of 20 statistically significant networks for the P5 vs P7 gene list, and 21 for the P5 vs P6 list were generated this way. IPA calculated a score for each network based on the number of connections between the molecules and how likely the molecules are together by chance, so that the higher the score, the more relevant the network is to the gene list. The networks shown here are merged from 3 top-scoring networks for P5 vs P6 (upper panel) and P5 vs P7 (lower panel). The lines between genes represent known direct (solid) or indirect (dashed) interactions. The coloured shapes represent up-regulated (red) or down-regulated (green) genes, with the intensity of the colour proportional to the fold change. The non-coloured shapes indicate genes that belong to the network in the Ingenuity knowledge base but were not picked up by our list.
(1.45 MB PDF)

## References

[1] Chisari FV (2005). Unscrambling hepatitis C virus-host interactions.. Nature.

[2] Semmo N, Day CL, Ward SM, Lucas M, Harcourt G (2005). Preferential loss of IL-2-secreting CD4+ T helper cells in chronic HCV infection.. Hepatology.

[3] Urbani S, Boni C, Missale G, Elia G, Cavallo C (2002). Virus-specific CD8+ lymphocytes share the same effector-memory phenotype but exhibit functional differences in acute hepatitis B and C. J Virol.

[4] Gale M, Foy EM (2005). Evasion of intracellular host defence by hepatitis C virus.. Nature.

[5] MacDonald AJ, Duffy M, Brady MT, McKiernan S, Hall W (2002). CD4 T helper type 1 and regulatory T cells induced against the same epitopes on the core protein in hepatitis C virus-infected persons.. J Infect Dis.

[6] Billerbeck E, Bottler T, Thimme R (2007). Regulatory T cells in viral hepatitis.. World J Gastroenterol.

[7] Cabrera R, Tu Z, Xu Y, Firpi RJ, Rosen HR (2004). An immunomodulatory role for CD4(+)CD25(+) regulatory T lymphocytes in hepatitis C virus infection.. Hepatology.

[8] Rushbrook SM, Ward SM, Unitt E, Vowler SL, Lucas M (2005). Regulatory T cells suppress in vitro proliferation of virus-specific CD8+ T cells during persistent hepatitis C virus infection.. J Virol.

[9] Boettler T, Spangenberg HC, Neumann-Haefelin C, Panther E, Urbani S (2005). T cells with a CD4+CD25+ regulatory phenotype suppress in vitro proliferation of virus-specific CD8+ T cells during chronic hepatitis C virus infection.. J Virol.

[10] Smyk-Pearson S, Golden-Mason L, Klarquist J, Burton JR, Tester IA (2008). Functional suppression by FoxP3+CD4+CD25(high) regulatory T cells during acute hepatitis C virus infection.. J Infect Dis.

[11] Fontenot JD, Rudensky AY (2005). A well adapted regulatory contrivance: regulatory T cell development and the forkhead family transcription factor Foxp3.. Nat Immunol.

[12] Li S, Gowans EJ, Chougnet C, Plebanski M, Dittmer U (2008). Natural regulatory T cells and persistent viral infection.. J Virol.

[13] Sugimoto K, Ikeda F, Stadanlick J, Nunes FA, Alter HJ (2003). Suppression of HCV-specific T cells without differential hierarchy demonstrated ex vivo in persistent HCV infection.. Hepatology.

[14] Li S, Jones KL, Woollard DJ, Dromey J, Paukovics G (2007). Defining target antigens for CD25+ FOXP3 + IFN-gamma- regulatory T cells in chronic hepatitis C virus infection.. Immunol Cell Biol.

[15] Allan SE, Crome SQ, Crellin NK, Passerini L, Steiner TS (2007). Activation-induced FOXP3 in human T effector cells does not suppress proliferation or cytokine production.. Int Immunol.

[16] Gavin MA, Torgerson TR, Houston E, DeRoos P, Ho WY (2006). Single-cell analysis of normal and FOXP3-mutant human T cells: FOXP3 expression without regulatory T cell development.. Proc Natl Acad Sci U S A.

[17] Baron U, Floess S, Wieczorek G, Baumann K, Grutzkau A (2007). DNA demethylation in the human FOXP3 locus discriminates regulatory T cells from activated FOXP3(+) conventional T cells.. Eur J Immunol.

[18] Zheng Y, Josefowicz SZ, Kas A, Chu TT, Gavin MA (2007). Genome-wide analysis of Foxp3 target genes in developing and mature regulatory T cells.. Nature.

[19] Dinarello CA, Kim SH (2006). IL-32, a novel cytokine with a possible role in disease.. Ann Rheum Dis.

[20] Ward S, Lauer G, Isba R, Walker B, Klenerman P (2002). Cellular immune responses against hepatitis C virus: the evidence base 2002.. Clin Exp Immunol.

[21] Polansky JK, Kretschmer K, Freyer J, Floess S, Garbe A (2008). DNA methylation controls Foxp3 gene expression.. Eur J Immunol.

[22] Vukmanovic-Stejic M, Zhang Y, Cook JE, Fletcher JM, McQuaid A (2006). Human CD4+ CD25hi Foxp3+ regulatory T cells are derived by rapid turnover of memory populations in vivo.. J Clin Invest.

[23] Kasow KA, Chen X, Knowles J, Wichlan D, Handgretinger R (2004). Human CD4+CD25+ regulatory T cells share equally complex and comparable repertoires with CD4+CD25- counterparts.. J Immunol.

[24] Ward SM, Fox BC, Brown PJ, Worthington J, Fox SB (2007). Quantification and localisation of FOXP3+ T lymphocytes and relation to hepatic inflammation during chronic HCV infection.. J Hepatol.

[25] Manigold T, Shin EC, Mizukoshi E, Mihalik K, Murthy KK (2006). Foxp3+CD4+CD25+ T cells control virus-specific memory T cells in chimpanzees that recovered from hepatitis C.. Blood.

[26] Ebinuma H, Nakamoto N, Li Y, Price DA, Gostick E (2008). Identification and in vitro expansion of functional antigen-specific CD25+ FoxP3+ regulatory T cells in hepatitis C virus infection.. J Virol.

[27] Heeg MH, Ulsenheimer A, Gruner NH, Zachoval R, Jung MC (2009). FOXP3 Expression in Hepatitis C Virus-Specific CD4(+) T Cells During Acute Hepatitis C.. Gastroenterology.

[28] Floess S, Freyer J, Siewert C, Baron U, Olek S (2007). Epigenetic control of the foxp3 locus in regulatory T cells.. PLoS Biol.

[29] Jones KL, Brown LE, Eriksson EM, Ffrench RA, Latour PA (2008). Human dendritic cells pulsed with specific lipopeptides stimulate autologous antigen-specific T cells without the addition of exogenous maturation factors.. J Viral Hepat.

[30] Witt CS, Price P, Kaur G, Cheong K, Kanga U (2002). Common HLA-B8-DR3 haplotype in Northern India is different from that found in Europe.. Tissue Antigens.

