# Correction: Analysis of FOXP3^+^ Regulatory T Cells That Display Apparent Viral Antigen Specificity during Chronic Hepatitis C Virus Infection

**DOI:** 10.1371/annotation/523d94fe-9730-499b-95b1-addd94089ff8

**Published:** 2012-06-22

**Authors:** Shuo Li, Stefan Floess, Alf Hamann, Silvana Gaudieri, Andrew Lucas, Margaret Hellard, Stuart Roberts, Geza Paukovic, Magdalena Plebanski, Bruce E. Loveland, Campbell Aitken, Simon Barry, Louis Schofield, Eric J. Gowans

The authors would like to acknowledge Matt Ardito and Annie De Groot of EpiVax, Inc. (Providence, Rhode Island) for providing the predictions that were used to synthesize the tetramers. None of the authors are affiliated with EpiVax, Inc. Epivax, Inc. had no role in study design, data collection and analysis, decision to publish, or preparation of the manuscript, aside from the above-mentioned contribution. The authors have declared that no competing interests exist. 

