# Supplementary material for: Analysis of FOXP3+ Regulatory T Cells That Display Apparent Viral Antigen Specificity during Chronic Hepatitis C Virus Infection
Source: PLoS Pathog. 2009 Dec 24;5(12):e1000707. doi: 10.1371/journal.ppat.1000707 (PMC2791198; doi:10.1371/journal.ppat.1000707)
Supplement: Table S1 — Full list of differentially expressed genes (0.35 MB PDF) [file ppat.1000707.s001.pdf]

Table S1

Table S1-A (P5 vs.P6 and P5 vs. P7)

Filtered for differential P value (Diff Pval) <0.05 and fold change >1.5 (Log $_2$FC> 0.585 or <-0.585). The data space is empty if the criteria is not met.

All genes shown here have a detection P value <0.001 for at least P5

TargetID	SYMBOL	P5 AVG_Signal	P6 AVG_Signal	P7 AVG_Signal	P5/P6 Log$_2$FC	Diff Pval	P5/P7 Log$_2$FC	Diff Pval
ILMN_7395	ABCB1	26.987	226.998	523.773	-3.072	2.2E-34	-4.279	2.6E-34
ILMN_21502	ACVR1	1010.122	611.237	412.035			1.294	5.7E-05
ILMN_8067	ADA	620.405	707.342	1108.356			-0.837	1.2E-02
ILMN_30039	ADAM23	105.412	194.346	146.961	-0.883	4.6E-03		
ILMN_14814	ADAM28	23.215	71.479	18.265	-1.622	1.5E-02		
ILMN_16701	ADAMTS4	61.293	5.831	6.483	3.394	7.7E-09	3.241	7.5E-09
ILMN_19769	ADCY9	42.680	78.093	112.554			-1.399	1.4E-02
ILMN_2022	ADRB2	89.250	304.156	609.906	-1.769	9.6E-03	-2.773	3.2E-04
ILMN_25998	AFF3	67.119	127.172	26.986	-0.922	2.0E-02		
ILMN_24336	ALS2CR2	885.369	480.350	444.384	0.882	2.6E-03	0.994	1.0E-03
ILMN_23219	ALS2CR4	166.744	78.770	101.675	1.082	2.5E-04		
ILMN_23873	ANGPTL6	36.612	75.110	39.225	-1.037	3.7E-02		
ILMN_2714	ANK3	90.867	441.031	435.994	-2.279	2.4E-20	-2.262	9.2E-05
ILMN_28640	ANKS1B	60.952	14.877	13.087			2.220	4.6E-02
ILMN_22521	ANTXR2	882.080	646.424	408.099			1.112	8.4E-03
ILMN_14184	ANXA1	1397.191	3900.022	3152.149	-1.481	2.0E-06	-1.174	5.7E-03
ILMN_2504	AOAH	45.084	89.490	306.167			-2.764	8.3E-12
ILMN_568	AP3M2	1054.110	725.307	589.984			0.837	2.5E-02
ILMN_4001	APBA2	52.631	58.122	196.803			-1.903	1.9E-04
ILMN_18531	APOBEC3F	107.878	254.607	116.814	-1.239	5.8E-05		
ILMN_23870	APS	18.061	58.976	26.834	-1.707	1.0E-04		
ILMN_23772	ARHGAP24	32.515	115.706	19.310	-1.831	2.5E-03		
ILMN_17477	ARID5B	2315.718	1787.858	1423.483			0.702	3.1E-02
ILMN_15416	ARL4C	611.610	292.789	289.171	1.063	2.4E-02	1.081	1.9E-02
ILMN_24845	ATF7IP2	223.760	327.888	462.830			-1.049	1.5E-03
ILMN_10855	ATP1B1	320.125	149.218	107.504	1.101	3.3E-02	1.574	1.8E-03
ILMN_4348	AUTS2	77.138	488.209	450.798	-2.662	5.4E-12	-2.547	6.0E-10
ILMN_6524	AXUD1	2084.573	1172.676	879.474	0.830	1.7E-03	1.245	9.7E-07
ILMN_16050	BAIAP2L1	31.435	8.945	1.372			4.518	4.4E-02

ILMN_25922	BANK1	231.650	1617.786	212.382	-2.804	2.5E-10		
ILMN_3307	BATF	1160.755	652.561	567.300	0.831	2.0E-03	1.033	1.3E-04
ILMN_17359	BCL11A	72.844	293.373	77.555	-2.010	1.9E-06		
ILMN_25971	BCL11A	19.782	106.622	19.612	-2.430	8.3E-11		
ILMN_3868	BCL2	2399.929	983.714	504.923	1.287	5.7E-08	2.249	1.6E-16
ILMN_12148	BCL2L1	656.518	275.446	350.680	1.253	7.7E-05	0.905	9.4E-03
ILMN_540	BCL6	736.738	1018.739	1280.835			-0.798	4.5E-02
ILMN_26827	BCNP1	20.653	118.590	23.547	-2.522	2.9E-07		
ILMN_23075	BFSP2	43.592	11.260	4.754	1.953	1.1E-02	3.197	7.8E-05
ILMN_23414	BLK	120.499	684.435	142.636	-2.506	2.2E-34		
ILMN_10290	BLNK	84.320	405.177	67.087	-2.265	3.3E-03		
ILMN_19896	BLR1	386.195	1109.996	424.734	-1.523	1.7E-08		
ILMN_27589	BLR1	24.084	83.698	20.095	-1.797	1.6E-03		
ILMN_16249	BMF	78.692	161.601	63.255	-1.038	4.3E-02		
ILMN_7936	BRDG1	84.632	356.289	109.799	-2.074	1.6E-04		
ILMN_27215	BTG3	754.334	539.980	451.688			0.740	2.2E-02
ILMN_28021	BTK	432.607	1218.227	454.247	-1.494	1.6E-02		
ILMN_5713	BTLA	132.660	278.435	195.950	-1.070	5.4E-05		
ILMN_12188	BYSL	337.232	143.940	156.925	1.228	2.9E-03	1.104	1.3E-02
ILMN_17888	C10orf38	189.336	241.424	349.256			-0.883	1.1E-02
ILMN_9655	C11orf17	865.864	577.592	501.625			0.788	6.6E-03
ILMN_19053	C13orf18	111.484	440.985	69.378	-1.984	2.7E-04		
ILMN_21076	C14orf139	500.571	321.709	285.172			0.812	8.7E-03
ILMN_25974	C16orf30	554.999	839.867	1103.876			-0.992	7.6E-03
ILMN_981	C18orf25	1904.438	1271.382	1133.263			0.749	1.4E-02
ILMN_14223	C1GALT1C1	921.022	628.150	515.012			0.839	4.2E-03
ILMN_25687	C1orf142	449.546	271.429	301.524	0.728	1.6E-02		
ILMN_18969	C1orf26	318.606	176.780	166.012	0.850	4.7E-02	0.940	2.8E-02
ILMN_7678	C1orf59	835.054	1039.112	1409.976			-0.756	3.4E-02
ILMN_2205	C20orf3	675.016	721.862	1134.832			-0.749	3.9E-02
ILMN_16665	C20orf35	1934.274	1361.986	1074.225			0.848	2.9E-03
ILMN_7928	C20orf55	1507.233	861.961	846.011	0.806	2.4E-03	0.833	4.0E-03
ILMN_9603	C3orf18	282.539	139.366	144.891	1.020	4.9E-04	0.963	6.8E-03
ILMN_28059	C3orf54	101.032	294.075	88.584	-1.541	4.0E-09		
ILMN_14619	C3orf59	382.505	146.219	78.568	1.387	1.7E-02	2.283	1.4E-04
ILMN_1756	C6orf190	71.491	285.579	351.560	-1.998	6.4E-10	-2.298	2.1E-10

ILMN_13979	C8orf70	268.950	157.567	60.200			2.160	8.8E-03
ILMN_20760	C9orf28	109.389	144.935	310.407			-1.505	5.0E-06
ILMN_21803	C9orf45	159.616	342.099	255.117	-1.100	3.1E-02		
ILMN_11032	C9orf88	1198.180	850.808	540.058			1.150	3.3E-04
ILMN_14654	CASK	328.038	211.932	197.588			0.731	4.0E-02
ILMN_4819	CCL4L1	119.073	214.640	1169.111			-3.295	7.3E-03
ILMN_22732	CCL5	115.780	639.401	3487.436	-2.465	1.7E-06	-4.913	3.9E-09
ILMN_1503	CCND2	3221.285	1899.822	2167.338	0.762	7.2E-03		
ILMN_21335	CCR7	3351.368	4220.431	6375.991			-0.928	6.6E-03
ILMN_2004	CD19	389.462	2025.985	404.720	-2.379	1.4E-08		
ILMN_11761	CD22	16.443	160.891	37.130	-3.291	4.7E-05		
ILMN_20704	CD300A	211.507	500.373	723.394			-1.774	2.4E-04
ILMN_2685	CD40LG	148.371	308.007	291.398	-1.054	2.1E-03	-0.974	7.9E-03
ILMN_18297	CD55	1898.946	1370.573	1189.430			0.675	4.8E-02
ILMN_20590	CD72	161.493	656.378	241.558	-2.023	5.2E-03		
ILMN_137396	CD79A	472.711	2551.681	585.818	-2.432	1.7E-23		
ILMN_138839	CD79B	604.941	1728.919	471.350	-1.515	1.2E-05		
ILMN_139125	CD79B	646.847	2149.299	460.463	-1.732	1.1E-11		
ILMN_2358	CD8A	141.182	359.702	2515.448			-4.155	3.1E-06
ILMN_137981	CD8B1	24.935	59.207	430.105			-4.108	9.9E-03
ILMN_2809	CD96	1444.144	2038.883	2521.285			-0.804	4.1E-02
ILMN_21651	CEACAM1	400.972	57.111	28.525	2.812	6.9E-04	3.813	1.6E-04
ILMN_20142	CEACAM1	1736.097	361.545	168.155	2.264	3.6E-05	3.368	2.7E-07
ILMN_7509	CENPE	41.957	9.674	31.476	2.117	4.7E-02		
ILMN_29669	CHD7	42.585	97.725	75.004	-1.198	4.4E-04		
ILMN_16218	CHN2	12.134	28.467	53.191			-2.132	3.9E-04
ILMN_8537	CHST10	17.281	35.120	50.155			-1.537	2.8E-02
ILMN_10462	CISH	2648.860	399.216	246.998	2.730	3.1E-15	3.423	3.0E-18
ILMN_15271	CITED4	617.651	193.782	214.334	1.672	2.8E-04	1.527	7.0E-04
ILMN_10458	CLCF1	102.386	179.652	151.787	-0.811	1.9E-02		
ILMN_13868	CMAH	575.955	247.312	235.633	1.220	1.0E-02	1.289	5.9E-03
ILMN_2147	CMKOR1	257.637	136.867	119.983	0.913	1.5E-03	1.103	3.0E-04
ILMN_789	CMTM6	2415.089	1684.534	1258.523			0.940	4.9E-04
ILMN_22539	CNFN	14.616	63.488	28.886	-2.119	3.9E-02		
ILMN_12678	COBLL1	116.450	684.136	87.069	-2.555	2.5E-06		
ILMN_7401	COCH	31.539	182.060	19.780	-2.529	3.8E-04		

ILMN_26478	CORO1B	1902.500	895.478	682.291	1.087	1.8E-05	1.479	3.2E-09
ILMN_26147	CPNE5	19.767	128.656	20.980	-2.702	4.2E-07		
ILMN_22421	CR2	12.912	104.563	40.605	-3.018	2.7E-06		
ILMN_17274	CSNK1G2	418.041	512.891	707.272			-0.759	3.9E-02
ILMN_24458	CST7	423.099	352.305	1996.513			-2.238	1.3E-03
ILMN_138103	CTLA4	4317.317	1244.044	938.850	1.795	5.0E-03	2.201	1.3E-03
ILMN_9279	CTNNAL1	96.365	52.661	49.233			0.969	2.3E-02
ILMN_4961	CTSW	289.235	229.020	1019.696			-1.818	4.3E-03
ILMN_10449	CXXC5	389.546	1058.862	514.579	-1.443	6.5E-04		
ILMN_9883	CYB561	52.410	309.759	315.083	-2.563	2.2E-34	-2.588	4.1E-07
ILMN_28843	CYBASC3	816.654	1422.440	955.285	-0.801	8.2E-03		
ILMN_711	CYP2U1	74.603	128.818	120.466	-0.788	3.5E-02		
ILMN_6966	CYSLTR1	126.839	386.159	201.853	-1.606	7.5E-06		
ILMN_9299	D4S234E	65.091	520.106	964.760	-2.998	2.2E-34	-3.890	2.6E-34
ILMN_24457	DBN1	59.482	42.110	309.819			-2.381	2.8E-02
ILMN_25837	DERL3	21.462	53.089	20.892	-1.307	7.7E-03		
ILMN_22993	DOCK6	21.663	13.718	1.033			4.391	4.5E-02
ILMN_21820	DOK2	94.141	37.815	39.344	1.316	1.6E-04	1.259	7.6E-04
ILMN_137779	DOK2	661.790	327.899	297.466	1.013	9.0E-03	1.154	8.0E-05
ILMN_26881	DSCR1L2	267.136	147.704	130.317	0.855	7.6E-03	1.036	4.7E-04
ILMN_12340	DUSP16	582.527	485.759	309.691			0.911	2.9E-02
ILMN_11097	DUSP4	449.770	98.291	155.752	2.194	1.6E-02		
ILMN_17249	E2F5	214.253	554.619	179.544	-1.372	9.1E-03		
ILMN_7978	EAF1	119.225	80.234	49.393			1.271	1.7E-04
ILMN_27934	EBF	27.309	188.948	21.261	-2.791	2.5E-11		
ILMN_5986	EBI2	1666.472	3275.493	2138.783	-0.975	2.3E-03		
ILMN_29351	EDAR	107.653	103.508	228.887			-1.088	4.5E-03
ILMN_14235	EFHC1	15.339	41.250	33.163	-1.427	1.9E-02		
ILMN_22990	EIF2C4	39.358	77.625	87.477	-0.980	1.9E-02	-1.152	1.0E-02
ILMN_9781	ELOVL4	35.834	170.753	131.361	-2.253	1.1E-02	-1.874	4.2E-04
ILMN_19998	EOMES	59.598	204.715	2150.807			-5.173	2.7E-12
ILMN_21869	EPHA4	87.669	341.960	286.177	-1.964	1.3E-02		
ILMN_7247	ETV6	434.029	710.966	572.147	-0.712	3.7E-02		
ILMN_26505	EVI2B	1576.656	3188.820	2433.941	-1.016	3.6E-05		
ILMN_20896	EZH2	137.628	75.294	105.720	0.870	1.3E-02		
ILMN_25961	F2R	13.618	48.150	147.830	-1.822	7.8E-04	-3.440	1.7E-04

ILMN_18681	FADS3	192.083	471.912	176.653	-1.297	1.5E-07		
ILMN_25614	FAM112A	34.415	7.878	7.559	2.127	5.1E-03	2.187	6.5E-03
ILMN_10067	FAM13A1	269.720	61.702	38.131	2.128	2.7E-04	2.822	1.3E-05
ILMN_3091	FAM3C	106.179	235.167	158.383	-1.147	1.9E-05		
ILMN_7706	FAM46C	1443.884	1035.765	717.556			1.009	6.8E-03
ILMN_6949	FASLG	12.909	51.260	136.596	-1.989	2.7E-03	-3.403	1.5E-02
ILMN_12030	FCER2	26.535	143.727	68.539	-2.437	6.7E-04		
ILMN_139282	FCGR2B	33.118	112.853	28.499	-1.769	1.6E-02		
ILMN_8487	FCRL1	83.581	420.085	86.282	-2.329	2.5E-12		
ILMN_2802	FCRL2	16.337	49.370	9.840	-1.595	1.9E-02		
ILMN_2355	FCRL6	116.278	124.103	305.906			-1.396	6.1E-03
ILMN_2598	FCRLM1	472.591	2371.092	446.606	-2.327	1.8E-06		
ILMN_24322	FCRLM2	45.985	110.468	62.730	-1.264	1.6E-04		
ILMN_14451	FHL3	195.374	95.886	131.724	1.027	9.6E-03		
ILMN_25398	FLJ14213	31.327	112.436	260.761	-1.844	2.3E-02	-3.057	4.6E-02
ILMN_2666	FLJ33641	62.608	378.818	95.256	-2.597	1.3E-04		
ILMN_15357	FLJ35695	49.583	10.652	3.257	2.219	2.4E-02	3.928	2.1E-03
ILMN_28347	FLJ37440	527.901	393.289	291.138			0.859	1.8E-02
ILMN_4408	FLJ37464	230.245	82.819	97.984	1.475	6.7E-08	1.233	6.2E-04
ILMN_36156	FLJ40311	124.288	68.028	64.063	0.869	1.5E-02	0.956	1.4E-02
ILMN_4754	FLT3LG	863.438	384.326	391.812	1.168	2.5E-04	1.140	7.9E-04
ILMN_7622	FOXP3	83.501	22.307	18.534	1.904	3.7E-07	2.172	9.7E-08
ILMN_3766	FXYD2	80.305	17.702	22.227	2.182	1.4E-04	1.853	1.7E-03
ILMN_21207	GAB3	66.206	176.650	230.853	-1.416	8.9E-07	-1.802	3.1E-06
ILMN_17355	GADD45A	416.398	196.389	93.740			2.151	2.7E-02
ILMN_8597	GALNAC4S-6ST	156.534	485.331	229.374	-1.632	4.7E-02		
ILMN_21972	GATM	19.147	85.224	27.542	-2.154	3.8E-04		
ILMN_18761	GCAT	25.190	41.955	66.557			-1.402	1.5E-02
ILMN_21048	GCET2	37.428	94.239	99.362	-1.332	2.2E-04	-1.409	3.0E-02
ILMN_17617	GFOD1	94.510	192.829	225.115	-1.029	6.2E-03		
ILMN_17168	GGA2	767.765	1258.293	989.421	-0.713	1.3E-02		
ILMN_27010	GIMAP5	3036.203	4580.928	5970.573			-0.976	3.0E-03
ILMN_6869	GIMAP8	505.530	1111.909	1482.565	-1.137	3.3E-06	-1.552	2.0E-07
ILMN_14068	GJB6	84.942	11.622	7.329	2.870	2.8E-03	3.535	1.3E-03
ILMN_3517	GLIPR1	527.389	1040.979	769.796	-0.981	8.3E-04		
ILMN_20255	GMNN	63.602	42.849	28.289			1.169	2.2E-02

ILMN_5207	GNG2	583.155	353.657	338.814			0.783	4.1E-02
ILMN_22105	GNG7	123.657	593.738	232.581	-2.263	2.5E-09		
ILMN_25463	GNG8	155.735	30.855	61.391	2.336	9.1E-04		
ILMN_17459	GNLY	56.742	221.986	1458.938			-4.684	7.3E-03
ILMN_5374	GNPTAB	1022.734	628.733	756.443	0.702	1.7E-02		
ILMN_28767	GPHN	169.205	110.457	83.222			1.024	2.5E-02
ILMN_16884	GPR18	358.746	824.444	747.765	-1.200	7.5E-03		
ILMN_20675	GPR19	294.123	198.412	168.174			0.806	4.4E-02
ILMN_22986	GPR55	198.552	73.987	69.944	1.424	1.0E-06	1.505	2.6E-07
ILMN_9790	GRAMD3	351.428	125.787	201.191	1.482	1.5E-03		
ILMN_23069	GSTM2	82.198	84.824	182.836			-1.153	1.2E-02
ILMN_21648	GZMA	325.202	716.532	4536.577			-3.802	2.6E-34
ILMN_10868	GZMK	257.697	655.419	3450.359			-3.743	2.6E-34
ILMN_46681	HIP1R	360.747	589.609	602.086	-0.709	1.7E-02		
ILMN_23615	HIP1R	445.731	769.829	762.243	-0.788	1.0E-02		
ILMN_29690	HIPK2	355.197	521.250	692.749			-0.964	2.6E-03
ILMN_27857	HLA-DOA	263.381	775.537	387.178	-1.558	3.6E-02		
ILMN_7656	HLA-DOB	209.207	838.542	267.959	-2.003	3.3E-05		
ILMN_41173	HLA-DQA1	1878.223	4709.952	1831.013	-1.326	3.3E-02		
ILMN_1899	HOP	23.447	83.915	134.174	-1.840	1.6E-04	-2.517	6.3E-03
ILMN_27069	HPSE	222.473	116.931	88.342	0.928	1.5E-03	1.332	3.7E-06
ILMN_6623	HSPA1A	539.442	309.383	201.228	0.802	5.0E-02	1.423	5.2E-08
ILMN_12918	ICA1	484.600	281.076	307.219	0.786	9.3E-04	0.658	9.1E-09
ILMN_28481	ID2	601.663	828.027	1629.703			-1.438	8.2E-04
ILMN_15068	IFNAR2	1199.353	843.829	720.692			0.735	2.0E-02
ILMN_23930	IGJ	356.246	1475.186	651.562	-2.050	5.6E-03		
ILMN_32659	IGSF9B	140.843	55.994	71.609	1.331	2.9E-03		
ILMN_16803	IL15	87.404	279.734	188.994	-1.678	6.5E-04		
ILMN_16084	IL18RAP	349.082	349.348	1291.105			-1.887	1.0E-04
ILMN_24627	IL2RA	2103.915	180.697	154.578	3.541	4.0E-08	3.767	8.4E-08
ILMN_19931	IL2RB	5469.442	2362.487	3579.670	1.211	8.3E-06		
ILMN_3781	IL32	1983.743	1148.536	1044.043	0.788	3.6E-03	0.926	6.6E-04
ILMN_1393	IL7	42.009	35.923	1.282			5.034	1.0E-02
ILMN_137414	IL7R	2767.264	7596.756	8349.401	-1.457	1.9E-08	-1.593	3.3E-07
ILMN_10513	IL7R	81.223	279.894	294.977	-1.785	2.2E-02	-1.861	3.3E-02
ILMN_6860	INCA	86.600	36.286	16.404			2.400	2.2E-03

ILMN_22637	INPP4B	598.797	473.261	353.025			0.762	1.6E-02
ILMN_12414	IRF4	126.506	80.794	44.982			1.492	4.9E-04
ILMN_20480	IRF8	715.928	2409.573	926.898	-1.751	2.2E-04		
ILMN_14503	IRS2	423.316	355.372	253.530			0.740	4.7E-02
ILMN_11930	KCNH3	86.361	35.526	48.517	1.281	2.0E-03		
ILMN_29015	KIAA0040	248.415	175.243	123.883			1.004	3.6E-02
ILMN_6408	KIAA0125	77.457	500.524	105.153	-2.692	8.3E-13		
ILMN_21473	KIAA0513	222.184	356.796	390.927	-0.683	3.6E-02	-0.815	2.5E-02
ILMN_5957	KIAA0746	1526.467	2442.800	1666.634	-0.678	1.9E-02		
ILMN_42090	KIAA1671	29.124	111.710	325.633	-1.939	2.3E-03	-3.483	2.3E-10
ILMN_19059	KIAA1913	31.097	183.224	110.754	-2.559	4.0E-05		
ILMN_21165	KLF11	171.917	126.448	92.699			0.891	1.0E-02
ILMN_24870	KLHL2	250.069	150.501	130.771	0.733	2.6E-02	0.935	2.8E-03
ILMN_25774	KLRB1	694.377	1770.898	1633.274	-1.351	1.7E-02		
ILMN_15508	KLRD1	29.333	95.885	819.386	-1.709	1.3E-02	-4.804	1.4E-07
ILMN_12613	KLRG1	221.787	345.786	788.889			-1.831	3.7E-06
ILMN_29874	KLRK1	44.965	202.701	1377.603	-2.172	2.2E-03	-4.937	2.6E-34
ILMN_10994	KMO	35.600	129.508	50.234	-1.863	8.8E-03		
ILMN_16684	KSP37	21.238	99.675	1178.639			-5.794	2.5E-02
ILMN_12588	LAMA5	30.744	238.345	36.816	-2.955	9.6E-07		
ILMN_13848	LAMC1	28.468	99.453	33.674	-1.805	3.9E-03		
ILMN_3642	LAMC3	215.758	38.851	50.677	2.473	5.2E-04	2.090	2.9E-03
ILMN_28310	LASS6	362.084	561.673	1018.568			-1.492	3.2E-05
ILMN_137018	LAT2	103.888	263.781	127.807	-1.344	1.1E-02		
ILMN_15062	LAYN	251.125	17.761	36.038	3.822	3.8E-05	2.801	6.6E-04
ILMN_6603	LIMA1	320.338	185.820	210.052	0.786	8.8E-03		
ILMN_18779	LIMS2	165.584	84.382	98.272	0.973	2.9E-03		
ILMN_20151	LMCD1	83.689	26.383	16.490	1.665	1.1E-03	2.343	6.6E-05
ILMN_5796	LMO4	333.435	899.909	572.852	-1.432	5.4E-05		
ILMN_37516	LOC144481	39.730	4.838	2.774	3.038	9.8E-03	3.840	5.4E-03
ILMN_11861	LOC152485	145.549	185.809	360.275			-1.308	6.2E-04
ILMN_6140	LOC201895	795.079	1464.354	859.757	-0.881	1.5E-03		
ILMN_12575	LOC285908	71.119	92.271	150.946			-1.086	2.3E-02
ILMN_37634	LOC338758	391.149	713.492	535.419	-0.867	3.6E-02		
ILMN_9164	LOC349136	19.799	93.946	90.443	-2.246	3.0E-03	-2.192	3.7E-06
ILMN_18133	LOC388692	59.185	82.998	147.066			-1.313	1.4E-02

ILMN_36916	LOC389599	105.579	49.240	44.609	1.100	2.2E-02	1.243	7.9E-03
ILMN_43464	LOC440667	599.238	977.945	977.952	-0.707	1.5E-02		
ILMN_33812	LOC440816	309.773	183.997	167.059	0.752	4.7E-02	0.891	1.3E-02
ILMN_44140	LOC642113	847.466	3243.221	1124.000	-1.936	1.7E-02		
ILMN_37967	LOC647450	442.663	1705.449	659.253	-1.946	2.3E-02		
ILMN_35193	LOC652314	41.043	14.782	2.766			3.891	2.1E-03
ILMN_35426	LOC652479	12.515	72.029	9.622	-2.525	1.9E-02		
ILMN_37502	LOC652493	650.502	2467.112	834.725	-1.923	9.1E-04		
ILMN_32875	LOC652694	121.374	611.588	212.278	-2.333	1.6E-02		
ILMN_39274	LOC652775	25.634	149.318	68.161	-2.542	5.8E-04		
ILMN_137005	LOC653613	62.883	6.370	4.445			3.822	4.6E-02
ILMN_10080	LOC90925	29.580	142.565	39.042	-2.269	4.6E-12		
ILMN_3858	LOC92691	87.236	48.302	41.288			1.079	8.2E-03
ILMN_12017	LPHN1	142.999	154.313	251.030			-0.812	3.9E-02
ILMN_9813	LRMP	535.489	1329.810	932.435	-1.312	1.1E-07		
ILMN_2030	LRP8	334.017	166.319	205.636	1.006	6.9E-04		
ILMN_3568	LRRC32	279.981	21.216	15.202	3.722	1.3E-04	4.203	1.0E-04
ILMN_3542	LTB	7414.759	5044.913	4244.387			0.805	9.4E-03
ILMN_10044	LXN	377.579	294.537	202.228			0.901	2.8E-03
ILMN_12433	LY86	381.458	1191.426	461.876	-1.643	8.2E-03		
ILMN_22187	LY9	937.597	1486.306	1741.077			-0.893	9.4E-03
ILMN_9328	MAD1L1	1183.890	724.938	672.193	0.708	1.5E-02	0.817	5.6E-03
ILMN_3411	MAL	900.405	535.857	578.129	0.749	3.7E-02		
ILMN_534	MAPK13	1293.200	848.932	794.722			0.702	3.3E-02
ILMN_6222	MCOLN2	304.204	874.736	794.687	-1.524	5.0E-05		
ILMN_19661	MEF2C	30.942	185.445	57.947	-2.583	1.2E-05		
ILMN_17595	MELL1	44.678	11.186	12.888	1.998	1.6E-03	1.794	3.1E-03
ILMN_15572	MGC15619	191.696	500.189	207.979	-1.384	3.0E-08		
ILMN_137903	MGC23909	49.120	41.293	19.760			1.314	3.3E-02
ILMN_28832	MGC33556	353.124	501.796	767.110			-1.119	4.2E-03
ILMN_15940	MGC33839	18.258	27.918	57.711			-1.660	3.1E-02
ILMN_38785	MGC39372	34.572	110.118	74.769	-1.671	2.4E-07		
ILMN_5164	MIB1	61.049	51.364	29.268			1.061	4.6E-02
ILMN_4207	MMD	792.435	451.935	403.776	0.810	2.8E-03	0.973	4.0E-04
ILMN_25898	MOSPD1	231.780	156.779	125.456			0.886	6.6E-03
ILMN_23782	MTHFD2	518.373	295.775	245.134	0.809	2.4E-02	1.080	1.2E-03

ILMN_7083	MTSS1	66.466	264.557	158.000	-1.993	8.3E-11	-1.249	4.2E-03
ILMN_19846	MUC1	651.773	219.583	188.373	1.570	7.5E-06	1.791	4.3E-06
ILMN_8912	MYO1G	2173.604	1276.744	1161.193	0.768	3.2E-02	0.904	1.2E-02
ILMN_20154	MYO5C	154.286	56.967	19.830			2.960	5.8E-05
ILMN_138327	NAPSB	129.559	610.797	93.287	-2.237	3.1E-03		
ILMN_13197	NCALD	170.874	144.440	325.372			-0.929	1.4E-02
ILMN_7892	NCF4	326.732	321.721	135.967			1.265	2.8E-02
ILMN_19702	NCR3	1637.706	830.480	997.091	0.980	3.3E-04		
ILMN_24220	NDRG1	1515.369	933.874	851.312	0.698	1.6E-02	0.832	4.0E-03
ILMN_28709	NELF	651.125	325.363	327.763	1.001	5.2E-04	0.990	5.2E-03
ILMN_26383	NELL2	96.512	1148.425	2711.836	-3.573	8.1E-08	-4.812	2.6E-34
ILMN_15943	NKG7	167.384	394.866	3950.502			-4.561	4.6E-03
ILMN_18267	NOS3	76.060	40.711	38.415			0.985	3.8E-02
ILMN_14871	NUDT8	232.655	130.687	151.443	0.832	6.4E-03		
ILMN_20212	OBFC2A	1417.505	1084.309	878.600			0.690	4.0E-02
ILMN_11112	OSBPL10	64.740	562.522	56.010	-3.119	8.3E-13		
ILMN_6779	OSBPL5	46.581	57.489	242.235			-2.379	2.4E-10
ILMN_13137	OSM	1499.826	503.158	250.030			2.585	3.7E-03
ILMN_1964	PACSIN1	17.042	15.753	56.380			-1.726	8.8E-03
ILMN_19873	PASK	470.772	1753.878	1823.818			-1.954	3.5E-02
ILMN_18537	PAWR	19.359	58.079	21.796	-1.585	3.5E-04		
ILMN_22596	PCAF	2698.441	1710.434	1606.503	0.658	3.0E-02	0.748	1.4E-02
ILMN_6597	PCSK5	105.869	123.154	199.565			-0.915	1.6E-02
ILMN_138594	PDE4B	443.996	271.518	167.920			1.403	2.6E-03
ILMN_6142	PDLIM1	56.836	377.810	104.721	-2.733	1.4E-12		
ILMN_11771	PELI1	632.486	374.773	264.614	0.755	8.5E-03	1.257	1.3E-06
ILMN_1531	PFN2	43.154	80.019	72.991	-0.891	8.5E-03		
ILMN_12419	PFTK1	70.899	247.885	57.268	-1.806	1.6E-02		
ILMN_13666	PHTF2	275.081	219.828	126.532			1.120	5.5E-03
ILMN_21964	PIM1	9363.224	5017.442	5291.682	0.900	2.8E-04	0.823	3.8E-03
ILMN_12660	PITPNC1	239.111	316.614	574.553			-1.265	1.5E-03
ILMN_17006	PKIG	30.051	96.609	26.376	-1.685	1.2E-04		
ILMN_3773	PLCG1	1371.776	883.455	1280.237	0.635	4.7E-02		
ILMN_27072	PLCG2	419.235	1277.263	517.028	-1.607	2.9E-03		
ILMN_16440	PLCL1	157.189	92.438	59.986			1.390	6.6E-05
ILMN_26768	PLEKHF2	225.943	408.518	181.658	-0.854	1.5E-02		

ILMN_28109	PLEKHG3	198.087	490.660	507.854	-1.309	6.1E-04	-1.358	8.0E-03
ILMN_28497	PLEKHK1	276.266	60.930	9.995	2.181	4.6E-02	4.789	1.8E-03
ILMN_23081	PLEKHO1	762.937	1198.029	907.412	-0.651	3.5E-02		
ILMN_1153	PLSCR3	235.410	147.629	133.242			0.821	1.8E-02
ILMN_8865	PLXDC1	22.800	54.001	104.773			-2.200	9.5E-03
ILMN_22628	PLXNB1	82.839	32.914	32.556			1.347	4.8E-02
ILMN_14800	PNOC	54.674	233.188	70.207	-2.093	5.4E-03		
ILMN_19978	POU2AF1	62.260	378.870	64.063	-2.605	1.3E-08		
ILMN_1833	PPAPDC1B	161.905	263.655	169.984	-0.704	3.6E-02		
ILMN_11092	PPM2C	564.982	408.943	293.474			0.945	2.6E-03
ILMN_9487	PPP1R3F	301.388	139.673	167.180	1.110	2.6E-05	0.850	7.6E-03
ILMN_22013	PRDM8	333.805	242.139	157.175			1.087	4.7E-03
ILMN_28175	PRF1	835.645	488.211	2574.397	0.775	1.3E-02		
ILMN_6622	PTGER2	935.362	441.205	253.778			1.882	2.8E-03
ILMN_20899	PTK2	105.981	305.048	227.004	-1.525	5.0E-03		
ILMN_137280	PTPLA	211.969	81.000	48.883	1.388	8.8E-03	2.116	5.2E-05
ILMN_11067	PTTG1	693.461	313.313	323.924	1.146	9.5E-03		
ILMN_10834	RAB11FIP1	212.095	114.783	99.488	0.886	1.1E-02	1.092	6.2E-04
ILMN_3988	RAB11FIP1	108.200	62.034	49.405			1.131	2.8E-03
ILMN_24051	RAB6IP1	609.538	1093.392	1291.272			-1.083	3.4E-03
ILMN_19038	RAG1AP1	2039.630	1185.735	885.948	0.783	3.3E-03	1.203	1.8E-06
ILMN_17213	RALGPS2	13.369	71.147	20.238	-2.412	3.9E-06		
ILMN_24486	RAPGEF3	18.990	53.642	20.883	-1.498	4.5E-02		
ILMN_14106	RASGEF1A	15.917	67.210	73.794	-2.078	3.6E-03	-2.213	3.8E-04
ILMN_25102	RBL2	1949.495	2335.487	3423.340			-0.812	4.2E-02
ILMN_18726	RBMS1	24.544	66.051	63.985	-1.428	4.5E-02		
ILMN_34353	RGAG4	27.020	80.471	56.260	-1.574	1.6E-05		
ILMN_16445	RGS16	373.577	313.446	118.526			1.656	7.8E-06
ILMN_14714	RHBDF2	576.530	1221.844	901.130	-1.084	1.8E-03		
ILMN_19962	RHPN2	29.800	69.887	46.803	-1.230	1.3E-02		
ILMN_7339	RNF157	201.137	108.719	146.151	0.888	2.6E-02		
ILMN_11976	SAMD3	253.728	257.494	794.714			-1.647	1.1E-02
ILMN_17752	SAMHD1	133.933	79.971	70.205			0.932	1.1E-02
ILMN_7778	SAMSN1	1203.507	1055.556	616.268			0.966	2.7E-02
ILMN_15731	SAV1	243.957	410.956	236.569	-0.752	1.1E-02		
ILMN_23730	SCRN1	106.616	277.258	161.908	-1.379	1.5E-03		

ILMN_138968	SDCCAG33	388.324	482.230	668.366			-0.783	2.9E-02
ILMN_17072	SEC31L2	388.214	200.929	338.401	0.950	4.1E-02		
ILMN_25026	SEMA4B	122.841	314.979	155.327	-1.358	1.3E-04		
ILMN_24615	SEMA4D	2629.743	3150.985	4375.370			-0.734	4.1E-02
ILMN_29190	SERINC5	22.463	36.798	76.544			-1.769	1.3E-02
ILMN_7089	SERPINI1	109.324	99.508	55.503			0.978	1.1E-02
ILMN_11604	SESN3	74.868	31.935	16.096	1.229	9.4E-03	2.218	2.2E-05
ILMN_4036	SETBP1	36.012	181.172	53.062	-2.331	4.8E-11		
ILMN_13165	SGCE	16.884	99.075	36.672	-2.553	3.3E-09		
ILMN_9531	SGPP1	2303.146	2087.099	1446.486			0.671	4.9E-02
ILMN_139087	SGPP2	237.224	165.090	81.625			1.539	2.4E-03
ILMN_19915	SHMT2	3298.744	2089.739	1530.581	0.659	2.9E-02	1.108	1.3E-05
ILMN_26592	SIRPB2	173.414	89.733	153.743	0.951	6.9E-03		
ILMN_3886	SIRPB2	554.485	319.851	486.282	0.794	7.3E-03		
ILMN_10688	SLC6A16	89.813	192.233	140.526	-1.098	9.3E-05		
ILMN_3183	SLCO4A1	263.230	116.114	80.224			1.714	1.6E-04
ILMN_17467	SLCO4C1	12.375	22.574	44.213			-1.837	9.5E-03
ILMN_8262	SMAD5	166.973	462.309	331.654	-1.469	4.0E-09	-0.990	3.2E-03
ILMN_20096	SMARCB1	413.490	679.030	417.395	-0.716	2.3E-02		
ILMN_4851	SOCS2	745.689	129.411	48.039	2.527	3.2E-02	3.956	6.7E-03
ILMN_20138	SOX13	42.691	119.968	151.786	-1.491	3.3E-03	-1.830	3.4E-03
ILMN_30180	SOX8	223.579	441.250	498.858			-1.158	1.4E-02
ILMN_8737	SPIB	108.451	492.504	75.242	-2.183	2.9E-07		
ILMN_1974	ST3GAL1	1021.391	1366.550	1761.170			-0.786	2.2E-02
ILMN_15305	ST6GALNAC2	30.481	70.797	56.876	-1.216	2.3E-02		
ILMN_2957	STAM	955.300	518.578	329.701	0.881	1.5E-03	1.535	1.7E-09
ILMN_1387	STAMBPL1	1683.040	1147.231	1038.900			0.696	3.5E-02
ILMN_136955	STAP2	47.876	15.942	25.607	1.586	4.2E-03		
ILMN_20263	STK17A	17.699	40.303	65.931			-1.897	1.4E-04
ILMN_17469	STOM	624.597	1222.299	1542.499			-1.304	1.0E-03
ILMN_19056	SUHW2	70.258	126.590	150.895	-0.849	4.3E-02	-1.103	6.8E-03
ILMN_25326	SUOX	634.326	253.340	200.136	1.324	1.0E-07	1.664	4.2E-09
ILMN_4104	SWAP70	421.860	952.082	248.930	-1.174	9.6E-03		
ILMN_30143	SYP	35.601	7.920	8.726	2.168	3.0E-02		
ILMN_20095	SYTL2	48.518	112.965	182.149	-1.219	1.2E-04	-1.909	3.8E-06
ILMN_25716	TARP	89.271	179.591	863.083			-3.273	1.0E-05

ILMN_25527	TBC1D9	40.037	196.758	20.292	-2.297	2.8E-06		
ILMN_27218	TCEA3	1432.904	863.794	805.868	0.730	1.7E-02	0.830	6.8E-03
ILMN_4452	TEAD2	23.074	78.434	10.159	-1.765	3.2E-02		
ILMN_19387	TFEB	26.194	57.265	27.154	-1.128	2.0E-02		
ILMN_22189	TGFBR2	835.850	1264.118	1499.335			-0.843	2.6E-02
ILMN_22620	TGFBR3	331.587	591.693	999.479			-1.592	1.5E-02
ILMN_17969	THOC3	92.175	71.589	39.361			1.228	2.6E-03
ILMN_12372	TJP3	703.640	379.508	323.927	0.891	3.2E-02	1.119	4.5E-03
ILMN_10669	TLE1	59.575	160.607	72.882	-1.431	2.2E-03		
ILMN_6615	TLR10	97.785	574.070	95.383	-2.554	1.4E-12		
ILMN_15638	TLR6	27.878	79.617	25.128	-1.514	1.7E-03		
ILMN_5498	TLR9	55.031	107.130	48.222	-0.961	1.1E-02		
ILMN_14479	TMEM110	70.115	39.296	28.132			1.318	1.1E-02
ILMN_4901	TMEM71	504.526	1107.549	1265.615	-1.134	2.5E-06	-1.327	6.9E-06
ILMN_13834	TMEPAI	63.832	131.555	96.891	-1.043	4.9E-02		
ILMN_1052	TMOD1	87.792	65.159	36.404			1.270	3.2E-02
ILMN_22155	TNFRSF13C	77.258	216.235	65.337	-1.485	4.3E-08		
ILMN_22838	TNFRSF18 (GITR)	226.473	36.019	41.881	2.652	2.7E-02		
ILMN_19893	TNFRSF1B	4965.262	2176.612	1882.186	1.190	4.3E-07	1.399	1.3E-07
ILMN_16587	TOX	91.169	42.160	52.684	1.113	6.2E-03		
ILMN_5543	TPD52L1	51.048	15.213	8.902	1.747	4.2E-04	2.520	6.9E-06
ILMN_5830	TRAF1	306.177	186.518	204.633	0.715	3.2E-02		
ILMN_17008	TSPAN13	31.437	241.065	59.248	-2.939	3.0E-12		
ILMN_2453	TSPAN3	664.798	1324.774	939.575	-0.995	1.2E-03		
ILMN_8032	TSPAN5	155.691	101.339	84.758			0.877	1.5E-02
ILMN_23448	TTC21A	54.436	121.934	70.153	-1.163	3.0E-02		
ILMN_12202	TUFT1	200.307	143.382	115.394			0.796	2.8E-02
ILMN_7381	TXLNB	28.930	67.462	19.694	-1.222	4.4E-02		
ILMN_20654	UAP1L1	435.389	246.951	298.003	0.818	1.3E-02		
ILMN_654	UBQLN2	1391.161	861.098	796.543			0.804	2.2E-02
ILMN_28400	UBTF	143.919	188.143	250.296			-0.798	4.7E-02
ILMN_24416	UGP2	650.271	379.066	300.876	0.779	1.4E-04	1.112	3.3E-04
ILMN_16188	UNQ5783	75.429	209.668	89.747	-1.475	1.3E-02		
ILMN_13470	UST	53.149	160.056	101.901	-1.590	9.1E-07		
ILMN_139375	VAMP4	286.505	212.564	164.379			0.802	9.9E-03
ILMN_14200	VAV2	63.061	157.943	77.025	-1.325	9.0E-06		

ILMN_22390	VAV3	394.062	216.238	137.878	0.866	3.2E-02	1.515	8.0E-05
ILMN_27565	VIPR1	123.700	219.391	308.326			-1.318	3.2E-02
ILMN_2270	VPREB3	289.723	2756.625	411.621	-3.250	1.3E-05		
ILMN_10614	WNT7A	34.934	88.386	82.773	-1.339	4.8E-02		
ILMN_17127	YWHAB	372.109	306.333	226.667			0.715	4.6E-02
ILMN_22581	ZDHHC23	137.128	116.777	66.846			1.037	3.5E-02
ILMN_5174	ZNF218	139.317	304.493	581.656	-1.128	2.3E-02		
ILMN_7933	ZNF342	323.639	665.965	345.301	-1.041	4.4E-03		
ILMN_28956	ZNF609	86.389	102.643	168.206			-0.961	1.4E-02
ILMN_2307	ZNF683	28.098	111.764	522.876			-4.218	6.6E-05
ILMN_21768	ZNFN1A4	684.776	107.764	68.731	2.668	7.1E-17	3.317	1.7E-20
ILMN_89369		428.991	81.612	86.186	2.394	1.1E-03	2.315	2.6E-03
ILMN_89918		67.414	30.060	31.831	1.165	6.8E-03	1.083	2.5E-02
ILMN_79580		75.103	35.288	33.027	1.090	7.9E-03	1.185	7.3E-03
ILMN_110599		107.271	58.002	45.223	0.887	3.3E-02	1.246	1.2E-03
ILMN_123683		216.277	124.280	170.747	0.799	1.7E-02		
ILMN_117456		220.643	391.772	232.045	-0.828	2.6E-02		
ILMN_128287		451.667	831.584	638.888	-0.881	4.1E-02		
ILMN_70828		189.395	408.752	271.045	-1.110	1.5E-05		
ILMN_71472		15.702	39.394	12.290	-1.327	4.7E-02		
ILMN_110801		18.388	52.841	33.685	-1.523	1.5E-02		
ILMN_71954		17.621	87.371	106.954	-2.310	2.6E-02	-2.602	3.7E-03
ILMN_73404		30.035	158.906	207.380	-2.403	8.3E-13	-2.788	1.7E-04
ILMN_84303		28.377	205.299	40.216	-2.855	2.2E-34		
ILMN_117157		44.151	343.718	359.786	-2.961	8.7E-04		
ILMN_80852		31.882	359.329	43.052	-3.494	2.2E-34		
ILMN_105242		76.575	7.761	1.781			5.426	4.6E-02
ILMN_96745		105.312	69.779	50.170			1.070	2.7E-02
ILMN_119658		220.424	169.551	127.045			0.795	4.6E-02
ILMN_70875		336.172	255.961	198.686			0.759	2.7E-02
ILMN_89485		271.323	424.699	507.637			-0.904	6.8E-03
ILMN_80172		26.665	53.085	70.807			-1.409	3.3E-03
ILMN_75484		14.815	27.329	45.654			-1.624	1.6E-02
ILMN_75987		37.577	86.182	121.672			-1.695	1.2E-03

Table S1-B (P6 vs P7)

Filtered for differential P value (Diff Pval) <0.05 and fold change >1.5 (Log2FC> 0.585 or <-0.585).

All genes shown here have a detection P value <0.001 for at least P6

TargetID	SYMBOL	5 AVG_Signa	6 AVG_Signa	7 AVG_Signa	P6/P7 Log_2FC	Diff Pval
ILMN_7395	ABCB1	26.98669	226.998	523.7731	-1.206	1.5E-04
ILMN_12727	ADAM19	1223.915	2034.971	1110.179	0.874	1.9E-02
ILMN_4121	ADAM28	9.586886	77.86831	14.32857	2.442	1.6E-02
ILMN_14814	ADAM28	23.21457	71.47896	18.26466	1.968	9.4E-03
ILMN_25998	AFF3	67.11906	127.1718	26.98569	2.237	2.0E-05
ILMN_2997	ALOX5	581.3074	1911.899	552.5931	1.791	3.0E-03
ILMN_2504	AOAH	45.08434	89.49037	306.1667	-1.775	3.3E-07
ILMN_4001	APBA2	52.63112	58.12161	196.8026	-1.760	2.8E-04
ILMN_23772	ARHGAP24	32.51532	115.7059	19.30997	2.583	7.4E-04
ILMN_14697	BACE2	33.57959	71.4373	14.65807	2.285	1.3E-05
ILMN_25922	BANK1	231.6503	1617.786	212.3819	2.929	3.3E-07
ILMN_17359	BCL11A	72.84441	293.3732	77.5554	1.919	6.8E-03
ILMN_25971	BCL11A	19.78169	106.6215	19.61187	2.443	6.3E-05
ILMN_3868	BCL2	2399.929	983.7138	504.923	0.962	4.9E-04
ILMN_26827	BCNP1	20.65281	118.59	23.54675	2.332	7.8E-04
ILMN_23414	BLK	120.4985	684.4351	142.6355	2.263	3.3E-07
ILMN_10290	BLNK	84.31991	405.1768	67.08655	2.594	6.1E-03
ILMN_27589	BLR1	24.08441	83.69763	20.09501	2.058	6.2E-04
ILMN_19896	BLR1	386.1952	1109.996	424.7338	1.386	4.4E-05
ILMN_16249	BMF	78.69151	161.6012	63.25476	1.353	6.1E-04
ILMN_31945	C10orf128	88.81499	109.9357	46.09138	1.254	3.3E-02
ILMN_19053	C13orf18	111.4838	440.9854	69.3783	2.668	3.7E-04
ILMN_28059	C3orf54	101.0316	294.0752	88.5838	1.731	1.3E-05
ILMN_13979	C8orf70	268.9497	157.5672	60.19978	1.388	6.3E-05
ILMN_20760	C9orf28	109.3889	144.9354	310.4065	-1.099	1.0E-03
ILMN_11754	CBFA2T3	35.00335	124.8248	17.98508	2.795	5.7E-05
ILMN_22732	CCL5	115.78	639.4009	3487.436	-2.447	8.8E-06
ILMN_10201	CCNG2	74.4697	91.63487	42.38627	1.112	9.5E-03
ILMN_2004	CD19	389.4615	2025.985	404.7204	2.324	1.6E-03

ILMN_25152	CD1C	56.95211	73.3205	15.21261	2.269	3.5E-02
ILMN_926	CD1D	64.92323	223.1746	38.3138	2.542	4.3E-02
ILMN_28723	CD24	32.7933	99.87142	9.332069	3.420	8.6E-03
ILMN_137396	CD79A	472.7108	2551.681	585.8177	2.123	4.8E-03
ILMN_138839	CD79B	604.9412	1728.919	471.3504	1.875	1.5E-03
ILMN_139125	CD79B	646.8466	2149.299	460.4628	2.223	7.7E-06
ILMN_24367	CD82	315.9751	563.6811	341.9156	0.721	4.6E-02
ILMN_24985	CD82	135.0997	235.3805	127.7666	0.881	4.5E-02
ILMN_27064	CD86	4.943338	52.9508	5.667865	3.224	6.8E-04
ILMN_2358	CD8A	141.1822	359.7022	2515.448	-2.806	1.1E-04
ILMN_137981	CD8B1	24.93475	59.20693	430.1052	-2.861	4.9E-02
ILMN_12678	COBLL1	116.4497	684.1362	87.06932	2.974	9.9E-05
ILMN_7401	COCH	31.53934	182.0603	19.7803	3.202	2.7E-04
ILMN_26147	CPNE5	19.76726	128.656	20.98038	2.616	6.1E-04
ILMN_24458	CST7	423.0988	352.305	1996.513	-2.503	4.8E-04
ILMN_4961	CTSW	289.2353	229.0201	1019.696	-2.155	1.1E-03
ILMN_2029	CXCR3	494.5414	354.5434	699.3088	-0.980	3.7E-03
ILMN_14583	CXCR6	234.8627	117.9262	248.904	-1.078	3.5E-02
ILMN_6966	CYSLTR1	126.8385	386.1591	201.8529	0.936	1.2E-02
ILMN_9299	D4S234E	65.09116	520.1062	964.7597	-0.891	1.6E-02
ILMN_24457	DBN1	59.48174	42.11021	309.8191	-2.879	1.5E-02
ILMN_13426	DCAL1	225.189	577.2856	111.6758	2.370	1.0E-03
ILMN_25837	DERL3	21.46237	53.08885	20.89221	1.345	4.9E-02
ILMN_11830	DKFZP434B03	510.2143	352.7026	607.1423	-0.784	4.6E-02
ILMN_28506	DKFZP564O0	2.908103	77.29057	9.465574	3.030	2.7E-03
ILMN_17249	E2F5	214.2527	554.6194	179.5441	1.627	2.0E-02
ILMN_27934	EBF	27.30872	188.9479	21.26083	3.152	7.6E-10
ILMN_29351	EDAR	107.6532	103.5076	228.8868	-1.145	1.2E-02
ILMN_14422	EDG8	3.49219	6.38123	43.557	-2.771	2.5E-02
ILMN_19998	EOMES	59.59779	204.7145	2150.807	-3.393	7.6E-10
ILMN_25961	F2R	13.61798	48.14991	147.8299	-1.618	4.9E-02
ILMN_18681	FADS3	192.0828	471.9116	176.6526	1.418	1.5E-07
ILMN_27581	FCGR2B	77.13184	243.749	56.11108	2.119	1.6E-02
ILMN_139282	FCGR2B	33.11807	112.8527	28.49879	1.985	1.6E-02
ILMN_8487	FCRL1	83.58086	420.0847	86.28168	2.284	6.8E-04
ILMN_2802	FCRL2	16.33736	49.3699	9.839579	2.327	8.5E-04

ILMN_2355	FCRL6	116.2781	124.1026	305.9058	-1.302	1.7E-02
ILMN_2598	FCRLM1	472.5913	2371.092	446.6064	2.408	2.2E-03
ILMN_625	FLJ33590	5.430062	10.78013	63.55027	-2.560	1.7E-02
ILMN_21972	GATM	19.14722	85.22402	27.54239	1.630	1.9E-02
ILMN_22105	GNG7	123.6574	593.7375	232.5811	1.352	5.0E-02
ILMN_6771	GPC2	333.8614	189.5135	362.3087	-0.935	4.9E-02
ILMN_13759	GPR30	1.901067	24.78376	3.28862	2.914	4.6E-02
ILMN_21648	GZMA	325.2017	716.5315	4536.577	-2.663	2.5E-10
ILMN_10868	GZMK	257.6969	655.4189	3450.359	-2.396	2.5E-10
ILMN_528	GZMM	494.6092	390.0401	851.1016	-1.126	4.1E-04
ILMN_7656	HLA-DOB	209.2071	838.5417	267.9594	1.646	1.8E-02
ILMN_9421	HRK	10.6406	29.77032	5.901351	2.335	1.6E-02
ILMN_18737	IER5	562.6078	712.2974	414.4996	0.781	3.8E-02
ILMN_16084	IL18RAP	349.082	349.3477	1291.105	-1.886	9.2E-08
ILMN_1393	IL7	42.00857	35.92274	1.281939	4.808	3.5E-05
ILMN_30033	ILDR1	4.280469	31.24882	1.00769	4.955	1.7E-04
ILMN_23329	KBTBD8	245.8141	317.452	154.312	1.041	1.0E-03
ILMN_6408	KIAA0125	77.45701	500.5241	105.1527	2.251	1.5E-04
ILMN_42090	KIAA1671	29.12393	111.7099	325.6332	-1.543	4.3E-04
ILMN_29548	KIR3DL2	9.417846	44.85507	155.0719	-1.790	4.6E-04
ILMN_22647	KLHL14	22.28907	55.75026	12.42314	2.166	1.6E-02
ILMN_15508	KLRD1	29.33331	95.88458	819.386	-3.095	8.2E-06
ILMN_3762	KLRD1	10.28247	48.4246	376.9945	-2.961	1.0E-07
ILMN_12613	KLRG1	221.7872	345.7864	788.8894	-1.190	4.1E-03
ILMN_29874	KLRK1	44.96463	202.7012	1377.603	-2.765	1.0E-12
ILMN_12588	LAMA5	30.7438	238.3451	36.81572	2.695	1.6E-04
ILMN_14813	LDLRAP1	861.4796	656.8682	1203.189	-0.873	4.5E-02
ILMN_11861	LOC152485	145.549	185.8089	360.2747	-0.955	3.2E-02
ILMN_43020	LOC642083	10.53132	21.54634	144.4884	-2.745	8.0E-03
ILMN_35426	LOC652479	12.51512	72.02941	9.622305	2.904	3.1E-02
ILMN_37887	LOC652495	12.43754	42.01048	6.665674	2.656	1.5E-02
ILMN_10080	LOC90925	29.57969	142.5646	39.04216	1.869	6.8E-03
ILMN_30563	LOC96597	10.70831	34.33628	5.253508	2.708	1.2E-02
ILMN_18317	LYL1	345.7633	492.0148	225.2681	1.127	6.1E-04
ILMN_25648	MOXD1	4.927917	45.36032	11.35672	1.998	3.4E-02
ILMN_16607	MRPL44	525.3169	650.6722	342.2065	0.927	4.0E-02

ILMN_138327	NAPSB	129.5593	610.7974	93.28719	2.711	1.4E-02
ILMN_13197	NCALD	170.8737	144.44	325.3719	-1.172	4.7E-03
ILMN_7892	NCF4	326.7323	321.7205	135.9671	1.243	3.1E-04
ILMN_22054	NEFL	13.89887	43.50558	7.510345	2.534	9.3E-04
ILMN_26383	NELL2	96.51234	1148.425	2711.836	-1.240	6.1E-04
ILMN_15943	NKG7	167.3843	394.8659	3950.502	-3.323	1.9E-02
ILMN_22430	NLGN2	118.4023	100.0605	189.4064	-0.921	4.0E-02
ILMN_11112	OSBPL10	64.73992	562.5221	56.01047	3.328	1.5E-09
ILMN_6779	OSBPL5	46.5806	57.48899	242.2347	-2.075	2.8E-08
ILMN_1964	PACSIN1	17.04206	15.75339	56.3797	-1.840	3.7E-03
ILMN_26264	PADI4	222.4703	95.70482	279.5522	-1.546	2.5E-02
ILMN_18537	PAWR	19.35922	58.07887	21.79588	1.414	5.0E-03
ILMN_12419	PFTK1	70.89871	247.8845	57.26787	2.114	4.9E-02
ILMN_3092	PHF16	67.89634	179.0865	41.50765	2.109	1.3E-02
ILMN_17006	PKIG	30.05081	96.6092	26.37622	1.873	2.7E-03
ILMN_26768	PLEKHF2	225.9431	408.5175	181.6576	1.169	7.4E-04
ILMN_5055	PLEKHG1	18.29797	37.7717	5.866124	2.687	2.1E-02
ILMN_19978	POU2AF1	62.26002	378.8703	64.0631	2.564	6.3E-05
ILMN_28175	PRF1	835.6448	488.2111	2574.397	-2.399	2.0E-02
ILMN_17213	RALGPS2	13.36852	71.14671	20.23835	1.814	5.4E-04
ILMN_24988	ROBO3	134.919	96.66065	197.3031	-1.029	1.7E-02
ILMN_5844	SAMD3	82.08922	72.65669	190.8041	-1.393	4.5E-02
ILMN_11976	SAMD3	253.7279	257.4944	794.7143	-1.626	6.9E-03
ILMN_15731	SAV1	243.9568	410.9555	236.5688	0.797	1.7E-02
ILMN_4036	SETBP1	36.01228	181.1719	53.06151	1.772	3.9E-04
ILMN_13165	SGCE	16.88373	99.07541	36.67199	1.434	3.8E-02
ILMN_139087	SGPP2	237.2235	165.0896	81.625	1.016	2.7E-02
ILMN_26163	SIGLEC10	33.44723	86.32544	21.42886	2.010	4.6E-02
ILMN_4948	SNX2	1303.572	1885.713	1135.909	0.731	2.8E-02
ILMN_10699	SNX22	11.30405	145.6247	25.90266	2.491	1.7E-03
ILMN_8737	SPIB	108.4513	492.5035	75.24216	2.711	1.9E-05
ILMN_20454	SSPN	0.4411635	30.1521	3.730882	3.015	2.7E-03
ILMN_4104	SWAP70	421.8596	952.0818	248.9297	1.935	3.7E-04
ILMN_25716	TARP	89.27134	179.591	863.0831	-2.265	5.2E-04
ILMN_25527	TBC1D9	40.03749	196.7576	20.29191	3.277	7.6E-08
ILMN_12126	TCF4	77.5225	224.8937	25.47723	3.142	1.0E-04

ILMN_4452	TEAD2	23.07381	78.43377	10.15872	2.949	2.8E-04
ILMN_6615	TLR10	97.78497	574.0702	95.3828	2.589	6.3E-05
ILMN_15638	TLR6	27.87836	79.6167	25.12817	1.664	1.2E-03
ILMN_9141	TNFRSF13B	291.9844	686.8109	58.93271	3.543	3.1E-04
ILMN_22155	TNFRSF13C	77.25778	216.2353	65.33711	1.727	1.5E-06
ILMN_17008	TSPAN13	31.43728	241.0647	59.24751	2.025	3.0E-02
ILMN_7381	TXLNB	28.92967	67.46201	19.69412	1.776	4.1E-03
ILMN_2270	VPREB3	289.7226	2756.625	411.621	2.744	6.9E-03
ILMN_2617	ZBTB32	158.2236	160.3056	47.38394	1.758	1.0E-03
ILMN_18125	ZNF532	67.67209	120.5137	36.58043	1.720	1.6E-04
ILMN_2307	ZNF683	28.09775	111.7637	522.876	-2.226	1.1E-02
ILMN_27976	ZP1	11.47074	30.71755	3.265282	3.234	1.7E-02
ILMN_71472		15.70207	39.39441	12.29034	1.680	2.6E-02
ILMN_113570		1.919014	23.98393	1.889118	3.666	2.2E-02
ILMN_125061		23.17259	48.20243	15.57411	1.630	7.8E-03
ILMN_84303		28.37741	205.2989	40.21601	2.352	2.2E-06
ILMN_80852		31.88198	359.3287	43.05152	3.061	5.4E-11
