# Supplementary material for: Analysis of FOXP3+ Regulatory T Cells That Display Apparent Viral Antigen Specificity during Chronic Hepatitis C Virus Infection
Source: PLoS Pathog. 2009 Dec 24;5(12):e1000707. doi: 10.1371/journal.ppat.1000707 (PMC2791198; doi:10.1371/journal.ppat.1000707)
Supplement: Table S2 — NS3 peptide array used in the current study (0.04 MB PDF) [file ppat.1000707.s002.pdf]

Table S2. NS3 peptide array used in the current study (supplied by BEI Resources, ATCC, Manassas Virginia, USA)

A) NS3 peptides of genotype 3a

ID	*Sequence*	*ID*	*Sequence*	*ID*	*Sequence*
1	APITAYAQQTRGLLGTIV	34	KVPAAYVAQGYNVLVL	67	CNVAVEQYVDFSLDPTF
2	AQQTRGLLGTIVTSLTGR	35	YVAQGYNVLVLNPSVAA	68	EQYVDFSLDPTFSIETCTA
3	LGTIVTSLTGRDKNVVA	36	NVLVLNPSVAATLGFGSF	69	DPTFSIETCTAPQDAVSR
4	SLTGRDKNVVAGEVQVL	37	SVAATLGFGSFMSRAYGI	70	TCTAPQDAVSRSQRRGR
5	KNVVAGEVQVLSTATQTF	38	FGSFMSRAYGIDPNIR	71	DAVSRSQRRGRTGRGRL
6	VQVLSTATQTFLGTTV	39	SRAYGIDPNIRTGNRTV	72	SQRRGRTGRGRLGTYRYV
7	TATQTFLGTTVGGVMWTV	40	DPNIRTGNRTVTTGAKL	73	GRGRLGTYRYVTPGER
8	GTTVGGVMWTVYHGAGSR	41	GNRTVTTGAKLTYSTYGK	74	GTYRYVTPGERPSGMF
9	MWTVYHGAGSRTLAGVKH	42	GAKLTYSTYGKFLAGGGC	75	VTPGERPSGMFDSVVL
10	AGSRTLAGVKHPALQMY	43	TYGKFLAGGGCSGGAYDV	76	RPSGMFDSVVLCECYDA
11	AGVKHPALQMYTNVDQDL	44	GGGCSGGAYDVIICDDCH	77	DSVVLCECYDAGCSWYDL
12	LQMYTNVDQDLVGWPA	45	AYDVIICDDCHAQDATSI	78	CYDAGCSWYDLQPAETTV
13	NVDQDLVGWPAPPGAKSL	46	DDCHAQDATSILGIGTVL	79	WYDLQPAETTVRLRAYL
14	GWPAPPGAKSLEPCTCGSA	47	ATSILGIGTVLDQAETA	80	AETTVRLRAYLSTPGLPV
15	KSLEPCTCGSADLYLVTR	48	IGTVLDQAETAGVRLTVL	81	RAYLSTPGLPVCQDHLDL
16	CGSADLYLVTRDADVIPA	49	AETAGVRLTVLATATPPG	82	GLPVCQDHLDLWESVF
17	LVTRDADVIPARRRGDSTA	50	LTVLATATPPGSITVPH	83	CQDHLDLWESVFTGLTHI
18	IPARRRGDSTASLLSPR	51	ATPPGSITVPHSNIEEVA	84	WESVFTGLTHIDAHFL
19	GDSTASLLSPRPLARLK	52	TVPHSNIEEVALGSEGEI	85	TGLTHIDAHFLSQTKQA
20	LLSPRPLARLKGSSGGPV	53	EEVALGSEGEIPFYGKAI	86	DAHFLSQTKQAGLNFSYL
21	ARLKGSSGGPVMCPSGHV	54	EGEIPFYGKAIPIACIK	87	TKQAGLNFSYLTAYQATV
22	GGPVMCPSGHVAGIFRAA	55	YGKAIPIACIKGGRHLIF	88	FSYLTAYQATVCARAQA
23	SGHVAGIFRAAVCTRGVA	56	ACIKGGRHLIFCHSKKK	89	YQATVCARAQAPPPSW
24	FRAAVCTRGVAKALQFI	57	RHLIFCHSKKKCDKMASK	90	CARAQAPPPSWDETWKCL
25	TRGVAKALQFIPVETL	58	SKKKCDKMASKLRGMGL	91	PPSWDETWKCLVRLKPTL
26	KALQFIPVETLSTQAR	59	KMASKLRGMGLNAVAYYR	92	WKCLVRLKPTLHGPTPLL
27	IPVETLSTQARSPSF	60	GMGLNAVAYYRGLDVSVI	93	KPTLHGPTPLLYRLGPV
28	TLSTQARSPSFSDNSTPPA	61	AYYRGLDVSVIPTTGDVV	94	PTPLLYRLGPVQNEICL
29	PSFSDNSTPPAVPQSYQV	62	VSVIPTTGDVVVCATDAL	95	RLGPVQNEICLTHPITKY
30	TPPAVPQSYQVGYLHA	63	GDVVVCATDALMTGF	96	EICLTHPITKYVMACMSA
31	PQSYQVGYLHAPTGSGK	64	VCATDALMTGFTGDFDSV	97	ITKYVMACMSADLEVTT
32	GYLHAPTGSGKSTKVPAA	65	MTGFTGDFDSVIDCNVAV		
33	GSGKSTKVPAAYVAQGY	66	FDSVIDCNVAVEQYVDF		

B) NS3 peptides of GT1a

ID	*Sequence*	*ID*	*Sequence*	*ID*	*Sequence*
1	DGMVSKGWRLLAPITAYA	*32*	AHLHAPTGSGKSTKVPAA	*63*	GFTGDFDSVIDCNTCVTQ
2	WRLLAPITAYAQQTRGLL	*33*	GSGKSTKVPAAYAAQGYK	*64*	SVIDCNTCVTQTVDFSLD
3	TAYAQQTRGLLGCIITSL	*34*	VPAAYAAQGYKVLVLNPS	*65*	CVTQTVDFSLDPTFTIET
4	RGLLGCIITSLTGRDKNQ	*35*	*QGYKVLVLNPSVAATLGF*	*66*	FSLDPTFTIETTTLPQDA
5	ITSLTGRDKNQVEGEVQI	*36*	*LNPSVAATLGFG*AYMSKA	*67*	TIETTTLPQDAVSRTQRR
6	DKNQVEGEVQIVSTATQT	*37*	TLGFGAYMSKAHGVDPNI	*68*	PQDAVSRTQRRGRTGRGK
7	EVQIVSTATQTFLAT	*38*	MSKAHGVDPNIRTGVRTI	*69*	TQRRGRTGRGKPGIYRFV
8	VSTATQTFLATCIN	*39*	DPNIRTGVRTITTGSPIT	*70*	GRGKPGIYRFVAPGERPS
9	ATQTFLATCINGVCWTVY	*40*	VRTITTGSPITYSTYGKF	*71*	YRFVAPGERPSGMFDSSV
10	TCINGVCWTVYHGAGTRT	*41*	SPITYSTYGKFLADGGCS	*72*	ERPSGMFDSSVLCECYDA
11	WTVYHGAGTRTIASPKGP	*42*	YGKFLADGGCSGGAYDII	*73*	DSSVLCECYDAGCAWYEL
12	GTRTIASPKGPVIQMYTN	*43*	GGCSGGAYDIIICDECHS	*74*	CYDAGCAWYELTPAE
13	PKGPVIQMYTNVDQDLVG	*44*	YDIIICDECHSTDATSIL	*75*	GCAWYELTPAETTV
14	MYTNVDQDLVGWPAPQGS	*45*	ECHSTDATSILGIGTVLD	*76*	WYELTPAETTVRLRAYMN
15	DLVGWPAPQGSRSLTPCT	*46*	TSILGIGTVLDQAETAGA	*77*	ETTVRLRAYMNTPGLPVC
16	PQGSRSLTPCTCGSSDLY	*47*	TVLDQAETAGARLVVLAT	*78*	AYMNTPGLPVCQDHLEFW
17	TPCTCGSSDLYLVTRHAD	*48*	TAGARLVVLATATPPGSV	*79*	LPVCQDHLEFWEGVFTGL
18	SDLYLVTRHADVIPVRRR	*49*	VLATATPPGSVTVSHPNI	*80*	LEFWEGVFTGLTHIDAHF
19	RHADVIPVRRRGDSRGSL	*50*	PGSVTVSHPNIEEVALST	*81*	FTGLTHIDAHFLSQTKQS
20	VRRRGDSRGSLLSPRPIS	*51*	HPNIEEVALSTTGEIPFY	*82*	DAHFLSQTKQSGENFPYL
21	RGSLLSPRPISYLKGSSG	*52*	ALSTTGEIPFYGKAIPLE	*83*	TKQ*SGENFPYLVAYQATV*
22	RPISYLKGSSGGPLLCPA	*53*	IPFYGKAIPLEVIKGGRH	*84*	*FPYLVAYQATVCARA*QAP
23	GSSGGPLLCPAGHAVGLF	*54*	IPLEVIKGGRHLIFCHSK	*85*	QATVCARAQAPPPSWDQM
24	LCPAGHAVGLFRAAVCTR	*55*	GGRHLIFCHSKKKCDELA	*86*	AQAPPPSWDQMWKCLIRL
25	VGLFRAAVCTRGVAKAVD	*56*	CHSKKKCDELAAKLVALG	*87*	WDQMWKCLIRLKPTLHGP
26	VCTRGVAKAVDFIPVENL	*57*	DELAAKLVALGINAVAYY	*88*	LIRLKPTLHGPTPLLYRL
27	KAVDFIPVENLETTMRSP	*58*	VALGINAVAYYRGLDVSV	*89*	LHGPTPLLYRLGAVQNEV
28	VENLETTMRSPVFTDNSS	*59*	VAYYRGLDVSVIPTSGDV	*90*	LYRLGAVQNEVTLTHPIT
29	MRSPVFTDNSSPPAVPQS	*60*	DVSVIPTSGDVVVVSTDA	*91*	QNEVTLTHPITKYIMTCM
30	DNSSPPAVPQSFQVAHLH	*61*	SGDVVVVSTDALMTGFTG	*92*	HPITKYIMTCMSADLEVV
31	VPQSFQVAHLHAPTGSGK	*62*	STDALMTGFTGDFDSVID	*93*	MTCMSADLEVVTSTWVLV
