# Supplementary material for: Analysis of FOXP3+ Regulatory T Cells That Display Apparent Viral Antigen Specificity during Chronic Hepatitis C Virus Infection
Source: PLoS Pathog. 2009 Dec 24;5(12):e1000707. doi: 10.1371/journal.ppat.1000707 (PMC2791198; doi:10.1371/journal.ppat.1000707)
Supplement: Table S3 — HLA typing data (0.07 MB PDF) [file ppat.1000707.s003.pdf]

Table S3. HLA typing data

Patient ID	*HLA type*
S07-04	A*0301 B*0702 B*2705 CW*0102 CW*0702 DRB1*0101 DRB1*1501
RW05	A*0222 A*3002 B*4701 B*5801 CW*0602 CW*0701 DRB1*0301 DRB1*1302
PH09	A*1101 A*6801 B*1302 B*4403 CW*0602 CW*1601 DRB1*0701 DRB1*1301
SA112	A*1102 A*2407 B*5401 CW*0102 DRB1*0901 DRB1*1201
RW19	A*0201 A*2902 B*4402 B*4403 CW*0501 CW*1601 DRB1*0701 DRB1*1301
SA11	A*0101 A*0301 B*0702 B*0801 CW*0701 CW*0702 DRB1*0301 DRB1*0401
PH22	A*1101 A*2601 B*4001 B*5501 CW*0303 CW*0304 DRB1*1101 DRB1*1103
PH08 gt2a	A*0301 B*0702 B*3906 CW*0702 DRB1*0101 DRB1*1302
