# Supplementary material for: Analysis of FOXP3+ Regulatory T Cells That Display Apparent Viral Antigen Specificity during Chronic Hepatitis C Virus Infection
Source: PLoS Pathog. 2009 Dec 24;5(12):e1000707. doi: 10.1371/journal.ppat.1000707 (PMC2791198; doi:10.1371/journal.ppat.1000707)
Supplement: Table S4 — NS3 T cell epitopes found in the literature. (0.08 MB PDF) [file ppat.1000707.s004.pdf]

Table S4. NS3 T cell epitopes found in the literature

A) T helper epitopes

Epitope	*Protein*	*Position*	*Genotype*	*MHC II Association*	*Reference*
TRTIASPKGPVIQMY	NS3	1087-1101	1A	class II	(4)
DLVGWPAPQGSRSLT	NS3	1107-1121	1A	class II	(4)
CPAGHAVGIFRAAVCTRGVA	NS3	1171-1190	1A	DRB1*0101, DRB1*0401, DRB1*1501,DRB1*0404	(23)
QGYKVLVLNPSVAAT	NS3	1247-1261	1A	DRB1*0101, DRB1*0401, DRB1*1001,DRB1*1104	(23)
GGRHLIFCHSKKKCD	NS3	1387-1401	1B	DR13,DR15	(9)
LIFCHSKKKCDELAA	NS3	1391-1405	1A	DRB1*15	(4)
HSKKKCDEL	NS3	1395-1403	1A/1B	DRB1*15	(4)
TQTVDFSLDPTFTIE	NS3	1459-1473	1B	DR13,DR15	(9)
EQYVDFSLDPTFSIE	NS3	1465-1479	3A	class II	(16)
MFDSSVLCECYDAGC	NS3	1511-1525	1A/1B	DR7, DR13	(9)
TTVRLRAYMNTPGLPV	NS3	1531-1550	1B	DRB1*0701	(23)
FPYLVAYQATVCARA	NS3	1583-1597	1B	DR13,DR15	(9)
TAYQATVCARAQAPP	NS3	1593-1607	3A	class II	(16)
YLVAYQATVCARAQAP	NS3	1585-1600	1B	DRB1*1001	(23)

B) CTL epitopes

Epitope	*Protein*	*Position*	*Genotype*	*MHC I Association*	*Reference*
AYSQQTRGL	NS3	1031-1039	1B	A24	(3)
VEGEVQIVSTAAQTF	NS3	1055-1069	1A	class I	(4)
CINGVWCTV	NS3	1073-1081	1A	A2	(5)
QTFLATCINGVCWTV	NS3	1067-1081	1A	class I	(4)
ATCINGVCWTVYHGAGTRTI	NS3	1071-1090	1A	class I	(6)
TRTIASPKGPVIQMY	NS3	1087-1101	1A	class I	(4)
AGTRTIASPKGPVIQM	NS3	1085-1100	1A	class I	(7)
MYTNVDQDL	NS3	1100-1108	1A	A24	(8)
RRRGDSRGSLLSPRP	NS3	1143-1157	1B	class I	(9)
LLCPAGHAV	NS3	1169-1177	1A	A2	(10)
CPAGHAVGIFRAAVCTRGVA	NS3	1171-1190	1A	A2	(6)
HAVGLFRAA	NS3	1175-1183	1A	A68	(6)
AYAAQGYKVL	NS3	1243-1252	1B	Cw*0303, Cw*0304	(11)
TLGFGAYMSK	NS3	1261-1270	1A	A11	(12)
GAYMSKAHGV	NS3	1265-1274	1A/1B	A11	(13)
LGFGAYMSK	NS3	1262-1270	1A	A3	(10)
TITTGAPVTYSTYGK	NS3	1284-1298	1B	A2	(14)
TGAPVTYSTY	NS3	1287-1296	1B	A2	(14)
TGSPITYSTY	NS3	1287-1296	1A	A2	(10)
TYSTYGKFL	NS3	1292-1300	1A/1B	A*2402	(15)
CHAQDATTVL	NS3	1318-1327	3A	B38	(16)
HPNIEEVAL	NS3	1359-1367	1A/1B	B*3501	(7)
GEIPFYGKAI	NS3	1376-1385	3A	B44	(17)
GEIPFYGKAI	NS3	1371-1380	1A	C*1402	(7)
FYGKAIPIEAI	NS3	1375-1385	1B	A*2402	(11)
HSKKKCDEL	NS3	1395-1403	1A/1B	B8	(4)
HSKRKCDEL	NS3	1395-1403	1A/1B	class I	(7)
HSKKKCDELT	NS3	1395-1404	1A	B8	(18)
ELAAKLVAL	NS3	1402-1410	1A	B8	(19)
ELAAKLVGL	NS3	1402-1410	1A	B8	(20)
LAAKLVALGINAVAY	NS3	1403-1417	1A	A2	(6)
ATDALMTCY	NS3	1436-1444	1A	A1	(21)
DVVVVATDALMTGYT	NS3	1431-1445	1A	class I	(4)
GDVVVVSTDALMTGY	NS3	1430-1444	1A	class I	(7)
ALMTGYT*GDFDSVI*	NS3	1439-1452	1A	class I	(7)
STDALMT*GFTGDFDS*	NS3	1436-1450	1A	class I	(22)
GFTGDFDSV	NS3	1443-1451	1A/1B	A*0206	(11)
CLIRLKPTLHGPTPLLYR	NS3	1610-1627	1A/1B	class I	(6)
LIRLKPTL	NS3	1611-1618	1A/1B	B8	(6)
TLTHPVTK	NS3	1636-1643	1A	A11	(12)
EVTLTHPITKYIMTCMSA	NS3	1634-1651	1A/1B	B8	(18)
TLTHPITK	NS3	1636-1643	1A/1B	A11	(18)

Supplementary references

1. Yamada, N., K. Tanihara, M. Mizokami, K. Ohba, A. Takada, M. Tsutsumi, and T. Date. 1994. Full-length sequence of the genome of hepatitis C virus type 3a: comparative study with different genotypes. *J Gen Virol* 75 (Pt 11):3279-3284.

2. Kolykhalov, A.A., E.V. Agapov, K.J. Blight, K. Mihalik, S.M. Feinstone, and C.M. Rice. 1997. Transmission of hepatitis C by intrahepatic inoculation with transcribed RNA. *Science* 277:570-574.

3. Kurokohchi, K., K. Arima, and M. Nishioka. 2001. A novel cytotoxic T-cell epitope presented by HLA-A24 molecule in hepatitis C virus infection. *J Hepatol* 34:930-935.

4. Wertheimer, A.M., C. Miner, D.M. Lewinsohn, A.W. Sasaki, E. Kaufman, and H.R. Rosen. 2003. Novel CD4+ and CD8+ T-cell determinants within the NS3 protein in subjects with spontaneously resolved HCV infection. *Hepatology* 37:577-589.

5. Cerny, A., P. Fowler, M.A. Brothers, M. Houghton, H.J. Schlicht, and F.V. Chisari. 1995. Induction in vitro of a primary human antiviral cytotoxic T cell response. *Eur J Immunol* 25:627-630.

6. Lauer, G.M., E. Barnes, M. Lucas, J. Timm, K. Ouchi, A.Y. Kim, C.L. Day, G.K. Robbins, D.R. Casson, M. Reiser, G. Dusheiko, T.M. Allen, R.T. Chung, B.D. Walker, and P. Klenerman. 2004. High resolution analysis of cellular immune responses in resolved and persistent hepatitis C virus infection. *Gastroenterology* 127:924-936.

7. Cox, A.L., T. Mosbruger, Q. Mao, Z. Liu, X.H. Wang, H.C. Yang, J. Sidney, A. Sette, D. Pardoll, D.L. Thomas, and S.C. Ray. 2005. Cellular immune selection with hepatitis C virus persistence in humans. *J Exp Med* 201:1741-1752.

8. Ito, A., T. Kanto, N. Kuzushita, T. Tatsumi, Y. Sugimoto, T. Miyagi, T. Takehara, K. Katayama, K. Mochizuki, N. Hiramatsu, A. Kasahara, I. Yoshiya, Y. Sasaki, M. Hori, and N. Hayashi. 2001. Generation of hepatitis C virus-specific cytotoxic T lymphocytes from healthy individuals with peptide-pulsed dendritic cells. *J Gastroenterol Hepatol* 16:309-316.

9. Dutoit, V., D. Ciuffreda, D. Comte, J.J. Gonvers, and G. Pantaleo. 2005. Differences in HCV-specific T cell responses between chronic HCV infection and HIV/HCV co-infection. *Eur J Immunol* 35:3493-3504.

10. Rosen, H.R., G. Marousek, and S. Chou. 2002. A longitudinal analysis of T-cell epitope-coding regions of hepatitis C virus after liver transplantation. *Transplantation* 74:209-216.

11. Hakamada, T., K. Funatsuki, H. Morita, T. Ugajin, I. Nakamura, H. Ishiko, Y. Matsuzaki, N. Tanaka, and M. Imawari. 2004. Identification of novel hepatitis C virus-specific cytotoxic T lymphocyte epitopes by ELISpot assay using peptides with human leukocyte antigen-A*2402-binding motifs. *J Gen Virol* 85:1521-1531.

12. Wong, D.K., D.D. Dudley, N.H. Afdhal, J. Dienstag, C.M. Rice, L. Wang, M. Houghton, B.D. Walker, and M.J. Koziel. 1998. Liver-derived CTL in hepatitis C virus infection: breadth and specificity of responses in a cohort of persons with chronic infection. *J Immunol* 160:1479-1488.

13. Cucchiarini, M., A.R. Kammer, B. Grabscheid, H.M. Diepolder, T.J. Gerlach, N. Gruner, T. Santantonio, J. Reichen, G.R. Pape, and A. Cerny. 2000. Vigorous peripheral blood cytotoxic T cell response during the acute phase of hepatitis C virus infection. *Cell Immunol* 203:111-123.

14. Kurokohchi, K., T. Akatsuka, C.D. Pendleton, A. Takamizawa, M. Nishioka, M. Battegay, S.M. Feinstone, and J.A. Berzofsky. 1996. Use of recombinant protein to identify a motif-negative human cytotoxic T-cell epitope presented by HLA-A2 in the hepatitis C virus NS3 region. *J Virol* 70:232-240.

15. Nakamoto, Y., S. Kaneko, H. Takizawa, Y. Kikumoto, M. Takano, Y. Himeda, and K. Kobayashi. 2003. Analysis of the CD8-positive T cell response in Japanese patients with chronic hepatitis C using HLA-A*2402 peptide tetramers. *J Med Virol* 70:51-61.

16. Guglietta, S., A.R. Garbuglia, V. Pacciani, C. Scotta, M.P. Perrone, L. Laurenti, E. Spada, A. Mele, M.R. Capobianchi, G. Taliani, A. Folgori, A. Vitelli, L. Ruggeri, A. Nicosia, E. Piccolella, and P. Del Porto. 2005. Positive selection of cytotoxic T lymphocyte escape variants during acute hepatitis C virus infection. *Eur J Immunol* 35:2627-2637.

17. Sidney, J., S. Southwood, V. Pasquetto, and A. Sette. 2003. Simultaneous prediction of binding capacity for multiple molecules of the HLA B44 supertype. *J Immunol* 171:5964-5974.

18. Timm, J., G.M. Lauer, D.G. Kavanagh, I. Sheridan, A.Y. Kim, M. Lucas, T. Pillay, K. Ouchi, L.L. Reyor, J. Schulze zur Wiesch, R.T. Gandhi, R.T. Chung, N. Bhardwaj, P. Klenerman, B.D. Walker, and T.M. Allen. 2004. CD8 epitope escape and reversion in acute HCV infection. *J Exp Med* 200:1593-1604.

19. Giuggio, V.M., H.L. Bonkovsky, J. Smith, and A.L. Rothman. 1998. Inefficient recognition of autologous viral sequences by intrahepatic hepatitis C virus-specific cytotoxic T lymphocytes in chronically infected subjects. *Virology* 251:132-140.

20. Ward, S., G. Lauer, R. Isba, B. Walker, and P. Klenerman. 2002. Cellular immune responses against hepatitis C virus: the evidence base 2002. *Clin Exp Immunol* 128:195-203.

21. Lauer, G.M., K. Ouchi, R.T. Chung, T.N. Nguyen, C.L. Day, D.R. Purkis, M. Reiser, A.Y. Kim, M. Lucas, P. Klenerman, and B.D. Walker. 2002. Comprehensive analysis of CD8(+)-T-cell responses against hepatitis C virus reveals multiple unpredicted specificities. *J Virol* 76:6104-6113.

22. Kennedy, P.T., S. Urbani, R.A. Moses, B. Amadei, P. Fisicaro, J. Lloyd, M.K. Maini, G. Dusheiko, C. Ferrari, and A. Bertoletti. 2006. The influence of T cell cross-reactivity on HCV-peptide specific human T cell response. *Hepatology* 43:602-611.

23. Schulze zur Wiesch, J., G.M. Lauer, C.L. Day, A.Y. Kim, K. Ouchi, J.E. Duncan, A.G. Wurcel, J. Timm, A.M. Jones, B. Mothe, T.M. Allen, B. McGovern, L. Lewis-Ximenez, J. Sidney, A. Sette, R.T. Chung, and B.D. Walker. 2005. Broad repertoire of the CD4+ Th cell response in spontaneously controlled hepatitis C virus infection includes dominant and highly promiscuous epitopes. *J Immunol* 175:3603-3613.
