# Supplementary figures and images for: Analysis of FOXP3+ Regulatory T Cells That Display Apparent Viral Antigen Specificity during Chronic Hepatitis C Virus Infection
Source: PLoS Pathog. 2009 Dec 24;5(12):e1000707. doi: 10.1371/journal.ppat.1000707 (PMC2791198; doi:10.1371/journal.ppat.1000707)

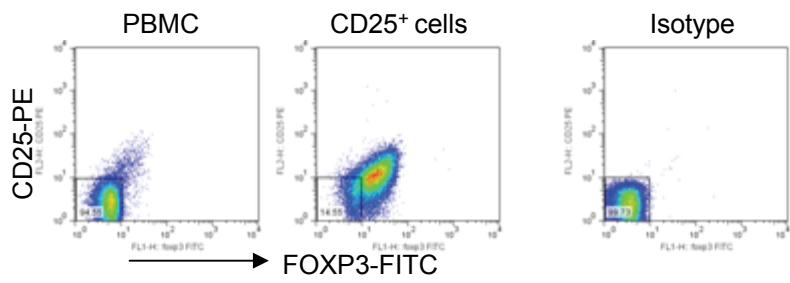

Supplement: Figure S1 — CD25 and FOXP3 expression profile of freshly isolated CD25+ cells. Freshly isolated CD25+ cells were stained for CD25 (surface) and FOXP3 (intracellular), followed by flow cytometry analysis gating on CD3+ lymphocytes (representative data from N = 3 HCV patients). (0.05 MB PDF) [file ppat.1000707.s005.pdf]

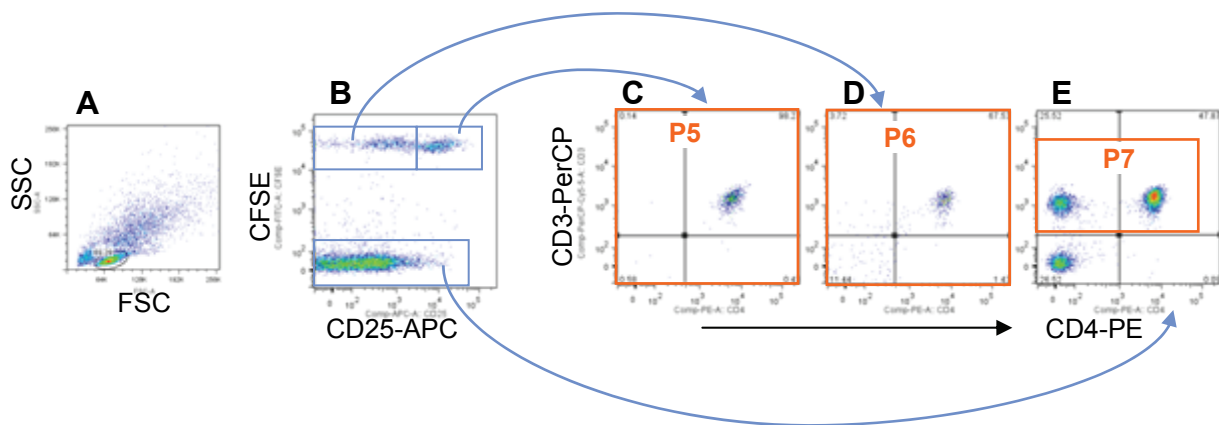

Supplement: Figure S2 — CD4 expression on day 5 of the co-culture. Plot (A) shows the lymphocyte gate, (B) shows the CD25 expression on the lymphocytes, (C) depicts CD4/CD3 expression on CFSE+CD25+ population (corresponding to P5, HCV-responsive Treg), (D) CD4/CD3 expression on CFSE-CD25+ population (corresponding to P6, HCV-non-responsive Treg) and (E) shows CD4/CD3 expression on CFSE unlabeled CD25 depleted fraction, the CD3+ cells are conventional T cells, corresponding to P7. (Representative data from N = 5 HCV patients). (0.08 MB PDF) [file ppat.1000707.s006.pdf]

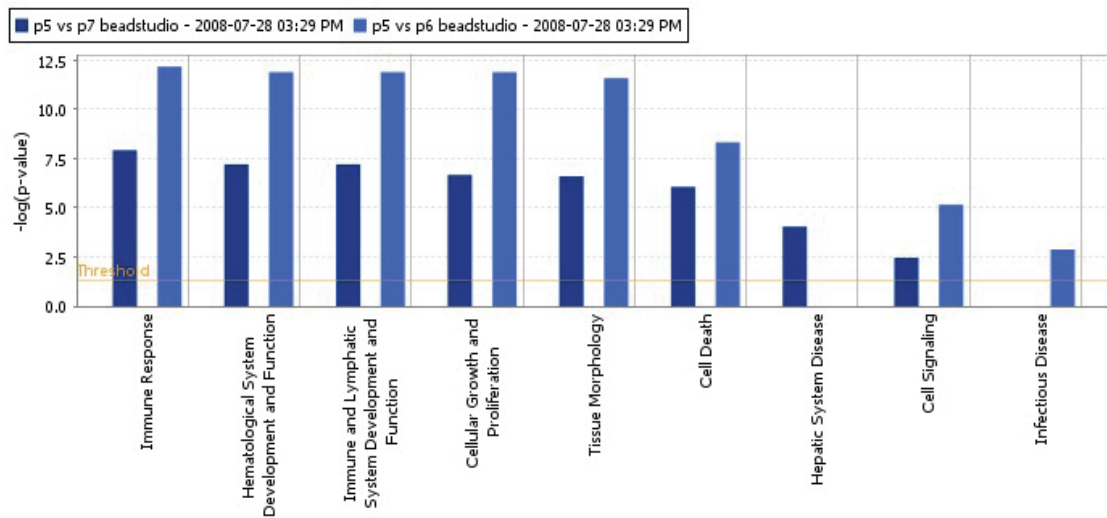

Supplement: Figure S3 — Ontology clustering using Ingenuity Pathway Analysis. The differentially expressed genes between P5 and P7 (dark blue) and between P5 and P6 (light blue bar) were grouped into 9 major functional clusters (x-axis). The y-axis indicates the Fisher's exact test P-value, that the higher the bar the less likely the genes might be found together owning to chance alone. (0.09 MB PDF) [file ppat.1000707.s007.pdf]

## P5 vs P6

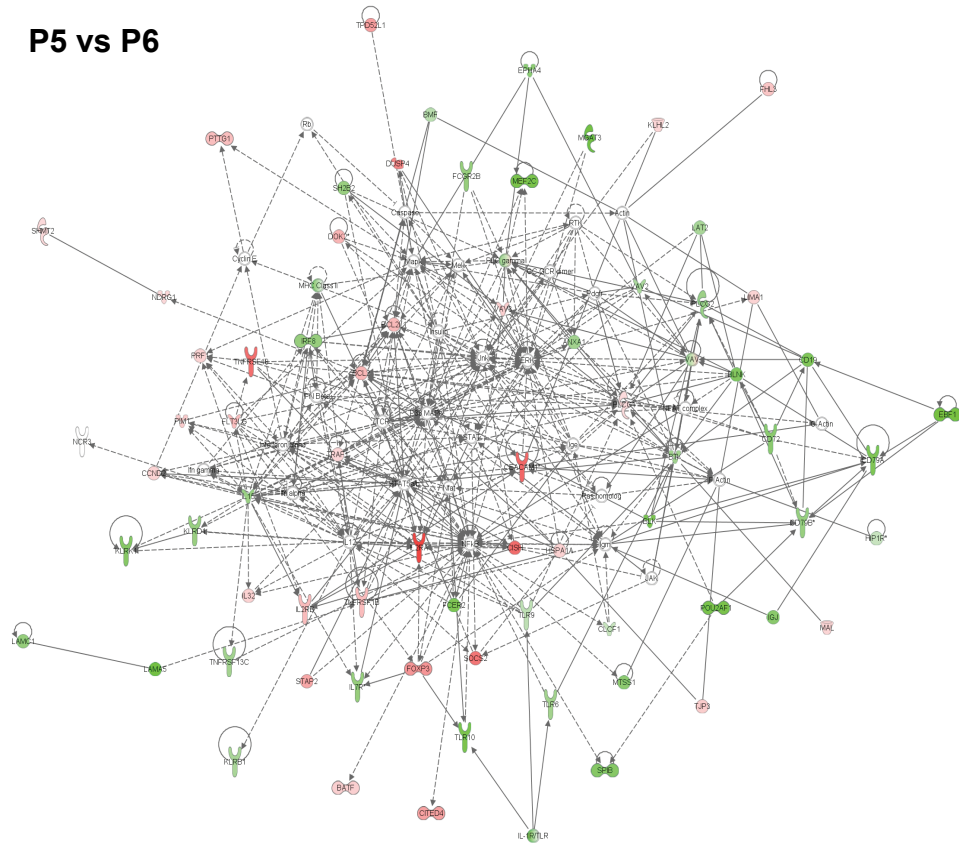

Supplement: Figure S4 — Network view illustrating relationships between differentially expressed genes. To construct networks, IPA overlays the differentially expressed genes with a literature based global molecular network, and identifies connections between these genes. A total of 20 statistically significant networks for the P5 vs P7 gene list, and 21 for the P5 vs P6 list were generated this way. IPA calculated a score for each network based on the number of connections between the molecules and how likely the molecules are together by chance, so that the higher the score, the more relevant the network is to the gene list. The networks shown here are merged from 3 top-scoring networks for P5 vs P6 (upper panel) and P5 vs P7 (lower panel). The lines between genes represent known direct (solid) or indirect (dashed) interactions. The coloured shapes represent up-regulated (red) or down-regulated (green) genes, with the intensity of the colour proportional to the fold change. The non-coloured shapes indicate genes that belong to the network in the Ingenuity knowledge base but were not picked up by our list. (1.45 MB PDF) [file ppat.1000707.s008.pdf]
